# Ancient DNA of Phoenician remains indicates discontinuity in the settlement history of Ibiza

**DOI:** 10.1038/s41598-018-35667-y

**Published:** 2018-12-04

**Authors:** Pierre Zalloua, Catherine J. Collins, Anna Gosling, Simone Andrea Biagini, Benjamí Costa, Olga Kardailsky, Lorenzo Nigro, Wissam Khalil, Francesc Calafell, Elizabeth Matisoo-Smith

**Affiliations:** 1 School of Medicine, Lebanese American University, Byblos, Lebanon; 2 Department of Anatomy, University of Otago, PO Box 56, Dunedin, 9054 New Zealand; 3 Department de Ciències Experimentals i de la Salut, Institute of Evolutionary Biology (CSIC-UPF), Universitat Pompeu Fabra, Barcelona, Spain; 4 Museu Arqueològic d’Eivissa i Formentera, Universitat de Barcelona, Illes Balears, Spain; 5 Facoltà di Lettere e Filosofia, Università di Roma, La Sapienza, Rome, Italy; 6 Department of Arts and Archaeology, Lebanese University, Beirut, Lebanon

## Abstract

Ibiza was permanently settled around the 7^th^ century BCE by founders arriving from west Phoenicia. The founding population grew significantly and reached its height during the 4^th^ century BCE. We obtained nine complete mitochondrial genomes from skeletal remains from two Punic necropoli in Ibiza and a Bronze Age site from Formentara. We also obtained low coverage (0.47X average depth) of the genome of one individual, directly dated to 361–178 cal BCE, from the Cas Molí site on Ibiza. We analysed and compared ancient DNA results with 18 new mitochondrial genomes from modern Ibizans to determine the ancestry of the founders of Ibiza. The mitochondrial results indicate a predominantly recent European maternal ancestry for the current Ibizan population while the whole genome data suggest a significant Eastern Mediterranean component. Our mitochondrial results suggest a genetic discontinuity between the early Phoenician settlers and the island’s modern inhabitants. Our data, while limited, suggest that the Eastern or North African influence in the Punic population of Ibiza was primarily male dominated.

## Introduction

The Phoenician culture was one of the most prevailing and widespread in the history of the Mediterranean basin. From its rise in the northern Levant, the Phoenicians connected east and west for over a millennium through their established trade networks across the Mediterranean, reaching beyond the Straits of Gibraltar. One of their first western outposts was the city of Gadir, modern Cadiz, on the Atlantic coast of Spain^1^ believed to be initially settled around 1100 BCE, then became a fully-fledged Phoenician settlement by the end of the 9^th^ century BCE^2^. Additional Phoenician settlements were established on the Balearic islands, Sardinia, Sicily, Malta, and Cyprus^2^. These Islands form a strategic arc across the northern Mediterranean allowing for island hopping from the Levantine homeland to the Iberian Peninsula and North African coast where they established their most dominant Western Mediterranean settlement in Carthage. The early Phoenician settlements in the Western Mediterranean are generally referred to as Western Phoenicia. From the middle of the sixth century BCE onwards, with the shift in Phoenician influence from the Levant to Carthage, they are typically referred to as Punic, and from the 6^th^ century BCE onward this term has become synonymous to Phoenicians outside the Levant. Our previous research has shown that the Phoenician settlers who encountered indigenous communities already living on the islands that they settled were integrated to form the new Phoenician societies^3^.

Archaeological evidence on the largest of the Balearic Islands, Mallorca and Menorca, indicates continuous settlement since the 3rd millennium BCE. Limited evidence favours some level of continuity of prehistoric settlement in Ibiza from the end of Bronze Age until the arrival of Phoenicians in the 7^th^ century BCE^4^. These Bronze Age settlements however, had relatively small populations compared to the larger Balearic Islands^5–7^. Ancient DNA analyses of human remains from early Ibizan sites can therefore provide evidence as to the origins of the Phoenician settlers of the island and the relationship between these early settlers and the modern population of the island. Further, when combined with other genetic data from Phoenician populations, analyses can provide key information about the process of Phoenician expansion and settlement of the Western Mediterranean.

The archaeological evidence to date suggests that the Phoenicians first settled on the island of Ibiza around 654 BCE^8^, arriving from west Phoenicia. They initially established a small settlement at the Bay of Ibiza and a second on Sa Caleta which was later abandoned. The first settlement gave rise to the Phoenician city of Ibusim or Ebusus^9^. The strategic importance of Ibiza did not escape the Phoenician navigators who realized that by controlling the Bay of Ibiza they could dominate maritime movement in all the North-western Mediterranean. Phoenician presence in these prime coastal locations asserted and ensured their maritime control over the entire Mediterranean and they controlled access to the Atlantic Ocean. For several centuries, the Phoenicians dominated metal trade from the Atlantic to the Eastern Mediterranean^2^.

The Phoenicians, and later the Punics, remained the main inhabitants of Ibiza for about seven centuries as demonstrated by the archaeological evidence from Puig des Molins, one of the largest necropoli in the Western Mediterranean, now a World Heritage site, with around 3000 tombs^10,11^. Burial data from this necropolis suggest three phases of the early demographic history of Ibiza^11^. The small founding population arrived from west Phoenicia, most probably Gadir, during the 7^th^ century BCE, and they are identified by their funerary rituals, that primarily involved cremation. The second phase occurred between the 5^th^ and 4^th^ centuries BCE and is indicated by a significant and rapid expansion in population size, reaching nearly 4,000 inhabitants. This growth may have been driven by a population flow from Carthage and other Punic settlements in the Mediterranean and coincides with a period of prosperity and major development of the island. The third and last phase of this early settlement period was between the 3^rd^ and 2^nd^ centuries during which Ibiza witnessed a period of economic decline and the return of cremation funerary rituals. After the 2^nd^ Punic War, Ibiza started a long process of integration in the Roman Empire, which culminated in 74 AD, when it became a *Latin Municipium*^12^. The Roman impact on the local inhabitants of Ibiza appears to have been minimal as is indicated by limited evidence of influence in the archaeological record. The Ibizan population remained somewhat isolated while under Roman political influence until the Islamic conquest of the island by Arabs and Berbers around 902 CE and they remained under Muslim rule for 333 years. Beginning around 1229 CE the Balearic Islands, including Ibiza in 1235, were impacted by immigrants from Catalonia, in mainland Spain, and the population of Ibiza slowly grew in number, but relative isolation of the autochthonous population continued and is still maintained today^4,13^. The extent of admixture between the indigenous Phoenician derived population of Ibiza and later occupiers has not been investigated at the genetic level, though genetic studies of the modern population of Ibiza indicate that they are distinct from the other Balearic Island and from mainland populations^13–16^. Pacelli and Márquez-Grant^17^ suggest that this difference may be due to the arrival of North African Punic settlers, a hypothesis that remains to be tested.

Here we report ancient DNA (aDNA) analyses of archaeological remains from two Punic necropoli on Ibiza, obtained through high-throughput sequencing. We determine the maternal ancestry of the founders of Ibiza through analyses of complete mitochondrial genomes (mitogenomes) of 8 archaeological samples from Ibiza. These ancient mitogenomes are compared to the first complete mitogenome data generated for 18 modern Ibizans to determine if there is genetic continuity between the original Phoenician settlers and the modern indigenous population of the island today and to elucidate the settlement history of Ibiza. The complete mitogenome from a Chalcolithic/Early Bronze-Age sample from the Island of Formentera was also obtained, providing evidence of the ancestry of the pre-Phoenician occupants of this island. Finally, we present the first genome-wide sequence data obtained from an ancient Phoenician and assess the likely ancestry of this individual from Ibiza. The accumulating aDNA evidence from Phoenicians from Ibiza, Sardinia, Tunisia and Lebanon is providing valuable information regarding the Phoenician expansion practices and the development of Phoenician communities in the Western Mediterranean.

## Results

### Samples processed

A total of 13 ancient tooth samples were obtained for aDNA analyses, from the Museu Arqueologic d’Elvissa i Formentera, in Ibiza, Spain: eleven samples from the urban Puig des Molins Necropolis, in the town of Eivissa^11,18,19^; one sample from the Cas Molí site, in Sant Antoni de Portmany^20^, also from the island of Ibiza; and one sample from the megalithic chamber tomb from the site of Ca na Costa, on Formentera^21^. The archaeological context for these samples is provided in the Supplemental Data 1. We were able to obtain data to reconstruct complete or near complete mitogenomes from nine of these samples and 0.47X average depth of coverage of the whole genome of the Cas Molí sample (MS10614).

A total of 18 modern DNA samples, obtained from volunteers from Ibiza, initially sampled by researchers from the Institut de Biologia Evolutiva (IBE), Barcelona, as part of a study on Y chromosomes and surnames, were sent to the University of Otago for full mitogenome sequencing. Individuals were only included in the sampling if all four grandparents were born on Ibiza and carried Ibizan surnames.

### Processing and sequencing of samples

All ancient DNA processing was conducted in a dedicated aDNA facility at the University of Otago. DNA was extracted from archaeological teeth using standard protocols^22^. Double stranded Illumina libraries were constructed with dual barcodes for the ancient samples as described previously^23^, and the mitochondrial DNA was captured using hybridization with modern human mitochondrial bait, generated in our lab^24^.

Modern DNA samples were sent by CSIC-UPF to the University of Otago where they were processed in a separate lab from the aDNA facility, and mitogenomes were amplified, prepared and sequenced as described previously^23^.

All ancient sequences obtained were assessed for potential contamination and DNA damage patterns. Deamination and fragment lengths were all as expected for ancient samples (Supplementary Data 2). Consensus sequences were created (including indels) and deposited in GenBank. Sequences were assigned to haplogroups using Haplogrep^25^ with Phylotree build 17^26^. All aDNA reads generated have been submitted to the NCBI sequence read archive identified by lab sample number. All mtDNA haplogroup determinations for samples processed in this study and associated archaeological site names, dates, GenBank accession numbers, coverage and contamination estimates are shown in Table 1. Additional sample information is provided in Supplementary Table 1. All ancient samples processed showed minimal estimates of contamination except for sample MS10589, from Formentara, which returned an estimate of 14%. We therefore filtered the data to only include damaged reads which resulted in reduced coverage but the same haplogroup call, so we included this pre-Phoenician sample in our analyses.

**Table 1:** Samples processed in this study with mtDNA haplogroup determinations, their associated archaeological site names, dates, GenBank accession numbers, coverage and contamination estimates.

Sample ID	Haplogroup	Site/Source Info	Age/Date	Coverage	Mean Depth	Contamination	Genbank #
Car256	L2c	Ibiza	Modern	100	482.8	—	MH043576
Eiv004	I1a1	Ibiza	Modern	97.4	162.6	—	MH043575
Eiv005	H7	Ibiza	Modern	100	316.2	—	MH043574
Eiv006	T1	Ibiza	Modern	100	524.6	—	MH043573
Eiv007	T2a1a	Ibiza	Modern	100	277.3	—	MH043572
Eiv008	V	Ibiza	Modern	99.7	295.4	—	MH043571
Eiv009	T2b28	Ibiza	Modern	100	126.7	—	MH043570
Eiv010	U2d	Ibiza	Modern	100	134.7	—	MH043569
Eiv011	T2a1a	Ibiza	Modern	100	345.1	—	MH043568
Eiv012	H13b1 + 200	Ibiza	Modern	100	310.7	—	MH043567
Eiv013	T2b28	Ibiza	Modern	100	289.6	—	MH043566
Eiv014	HV0 + 195	Ibiza	Modern	100	320.3	—	MH043565
Eiv015	T2b	Ibiza	Modern	100	275.4	—	MH043564
Eiv016	L2c	Ibiza	Modern	100	269.4	—	MH043563
Fer111	T2b3	Ibiza	Modern	100	54.1	—	MH043562
Gus203	K1a4a1	Ibiza	Modern	100	169.4	—	MH043561
Gus204	J2b1a	Ibiza	Modern	100	470.6	—	MH043560
Sla132	H14b	Ibiza	Modern	100	433.6	—	MH043559
MS10612	H3 + 152	Puig des Molins, Ibiza	3rd - 2nd c BCE	100	40.6547	0.0008941	MH043581
MS10613	U4a	Puig des Molins, Ibiza	4th c BCE	99.2	5.61681	0.0171749	MH043578
MS10614	T2b	Ca’s Moli, Ibiza	361–178 cal BCE	100	139.573	0.0044663	MH043577
MS10616	H1 + 152	Puig des Molins, Ibiza	Early Roman	99.9	18.6375	0.0174345	MH043580
MS10617	U5b3	Puig des Molins, Ibiza	5th - 4th c BCE	99.9	12.0362	0.0109457	MH043579
MS10619	H1c	Puig des Molins, Ibiza	5th - 4th c BCE	100	17.4834	0.0106004	MH043582
MS10620	J1c3g	Puig des Molins, Ibiza	4th c BCE	96.3	3.87096	0.0539312	MH043584
MS10622	H3	Puig des Molins, Ibiza	4th c BCE	100	29.3584	0.0038769	MH043583
MS10589	K1a1b1	Ca na Costa, Formentera	900–750 BCE	90.62	3.552	0.1401751	MH043585

### Generation of nuclear data

One ancient sample (MS10614) was selected for shotgun sequencing, based on the high genome coverage observed in the mitochondrial sequencing of this sample. Adequate preservation was confirmed with an estimated 9% endogenous DNA content. The sex of this sample was determined based on computing the number of alignments to the Y Chromosome (nY) as a fraction of the total alignments to both sex chromosomes (nX + nY)^27^. The ratio of Y:X + Y coverage was 0.0042 (SE 0.0001, 95% CI 0.0041–0.0044) and thus the individual was determined to be female, which is inconsistent with the sex determination based on skeletal characteristics. After mapping the shotgun reads to the UCSC reference genome hg19, the resulting bam file was aligned with the Human Origins SNP dataset to call a pseudo-haploid genotype^28^. This genotype was merged with previously published ancient^29,30^ and modern SNP data sets^31–33^, resulting in a total of 119,316 SNPs used in subsequent analyses (See Supplementary Table 2).

### Radiocarbon Dating of sample MS10614

A subsample of the tooth from which the DNA was extracted was sent to Beta Analytic Inc. (Miami) for AMS dating. It returned a conventional radiocarbon age of 2190 ± 30, which provided a calibrated date range of 361–178 cal BC (2310–2127 cal BP), at 95.4% probability. This date is consistent with the archaeological context proposed by the archaeologists who excavated the site, suggesting 3^rd^ century BCE.

#### Analysis of mitochondrial genomes

**Figure 1:**
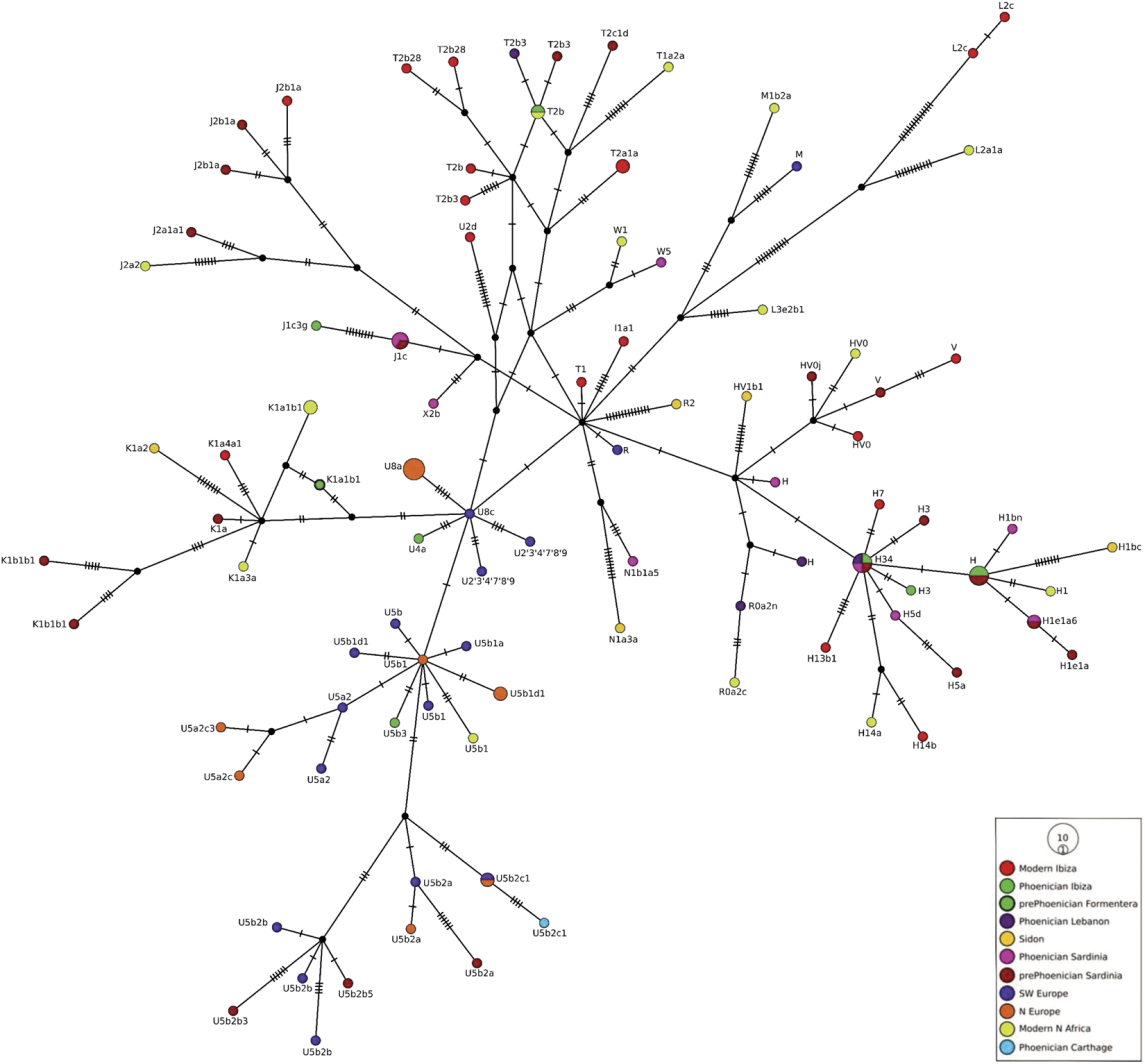
Median Joining (MJ) Network of complete mitogenomes from the ancient Phoenician and modern Ibizans and a Bronze Age Formentera sample generated in this study; ancient pre-Phoenicians from Sardinia^34^ and Sidon, Lebanon^29^, Phoenicians from Sardinia and Lebanon^3^ and Carthage^23^; ancient Southwestern Europeans from La Brana, Spain^35^, France and Italy^36^; ancient Northern Europeans (Germany)^36^ and modern North African populations^37^.

Nine complete mitogenomes were successfully obtained from 13 ancient samples and their Hg were determined. In addition, mitogenomes and their Hg were successfully determined from 18 modern Ibizans (Table 1). A Median Joining network of 111 mitogenomes representing the ancient and modern mitogenomes generated in this study with 83 publicly available ancient mitogenomes from European and Near Eastern^3,23,29,34–36^ and modern North African^37^ populations is shown in Fig. 1.

**Figure 2:**
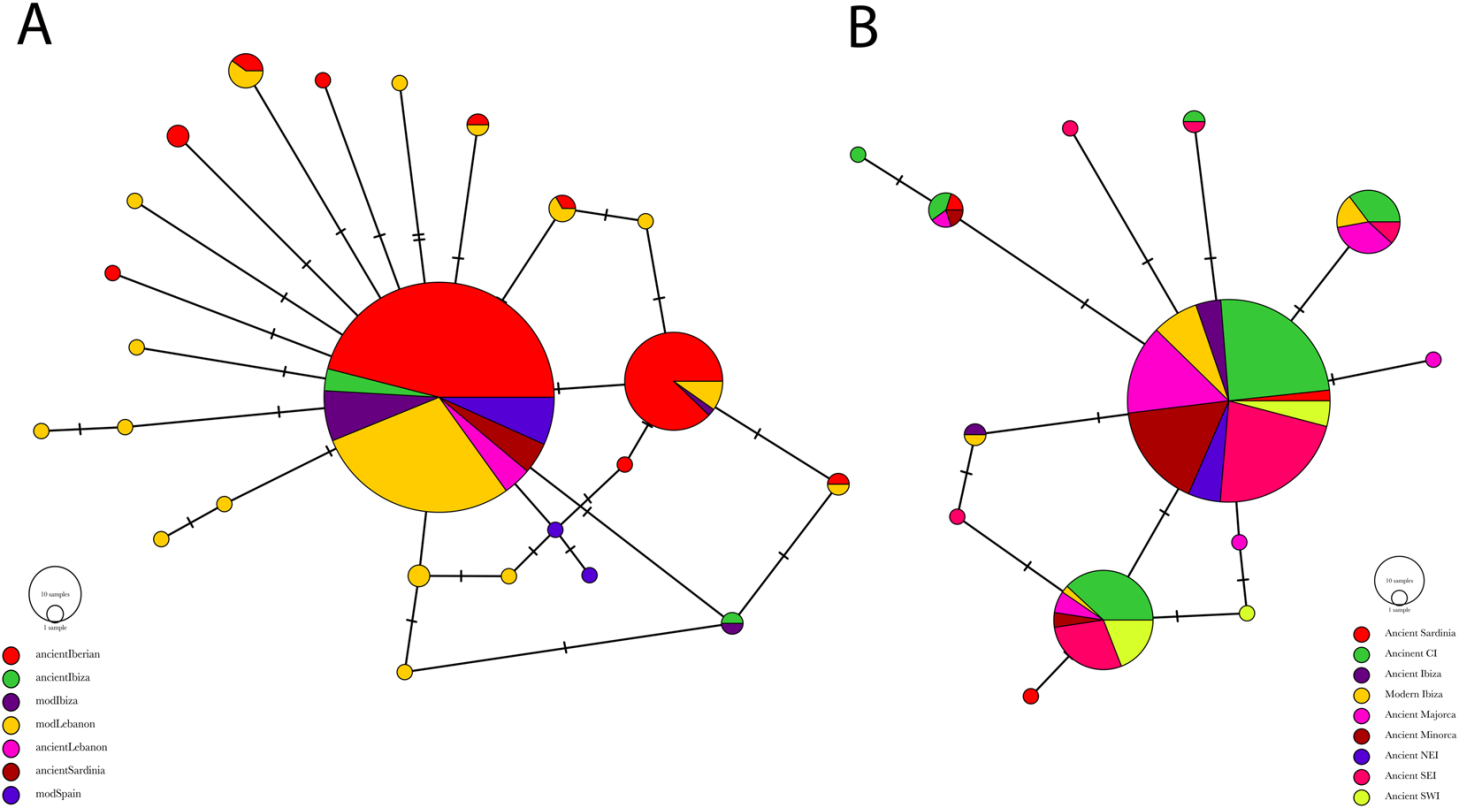
Median Joining (MJ) Networks for ancient and modern Ibizans with mtDNA HVR sequences of (**A**) 383 bp from Neolithic and Bronze Age Iberian samples (Ancient Iberians)^38^, modern Spanish^34^ Phoenician samples from Lebanon and Sardinia^3^ and (**B**) 156 bp of data from the Ancient Iberians and from Neolithic and Iron Aged samples from Mallorca and Menorca^39^.

To investigate the similarities between our Ibizans and other ancient samples from the Iberian mainland and the other Balearic islands we reduced our data to the comparable, publicly available HVR regions of 383 bp for Neolithic and Bronze age Iberian samples^38^ and 156 bp of data from Neolithic and Iron Age samples from Mallorca and Menorca^39^ and constructed Median Joining (MJ) networks shown in Fig. 2.

**Figure 3:**
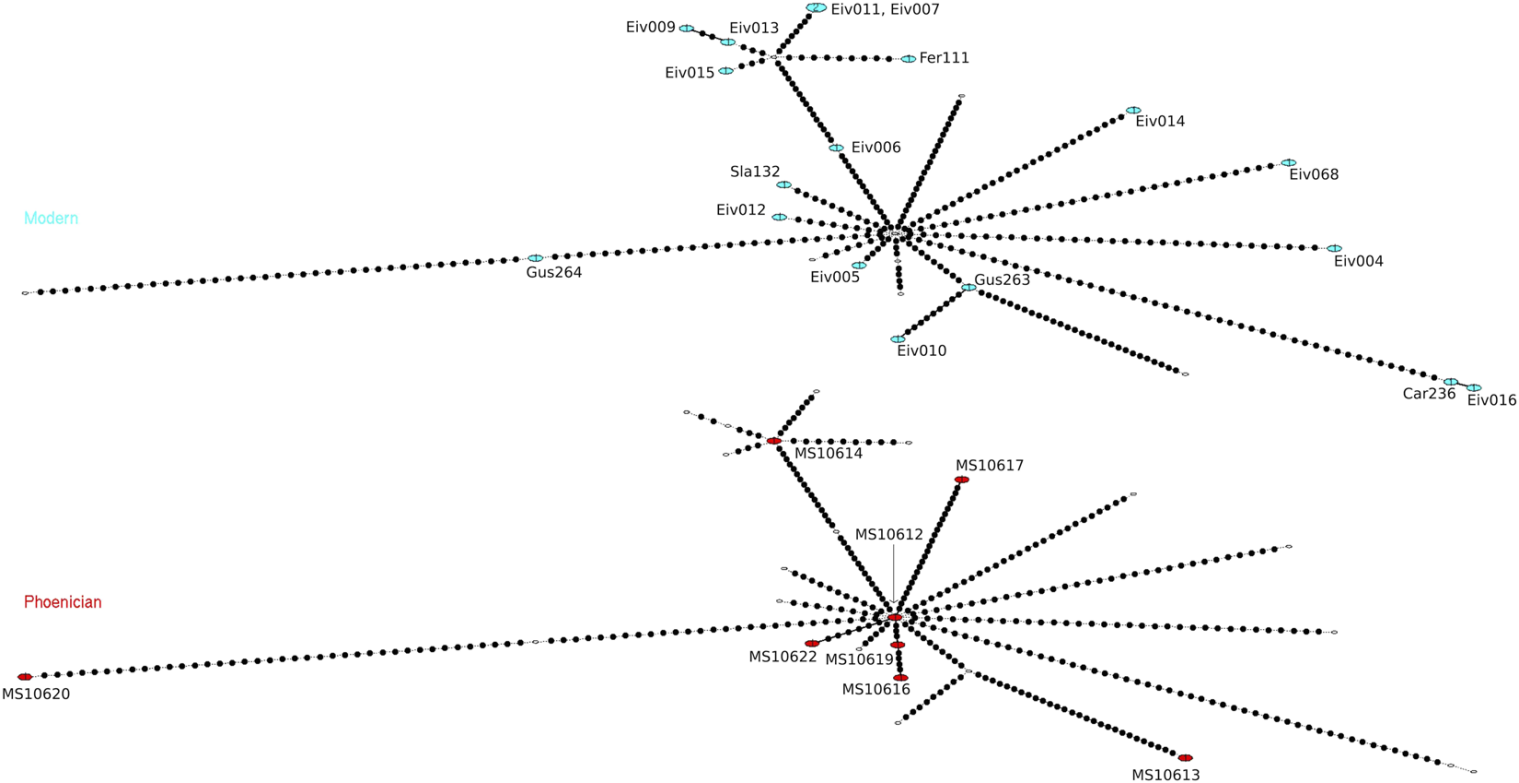
Temporal haplotype network constructed using ancient and modern Ibizan complete mitogenomes. Haplotypes are labelled by sample (Table 1). Numbers in circle indicates frequency of haplotype.

To test for continuity between ancient and modern Ibizan haplotypes, we performed a temporal network analysis^40^, shown in Fig. 3, which indicates that there were no shared haplotypes between the ancient and modern Ibizans.

**Figure 4:**
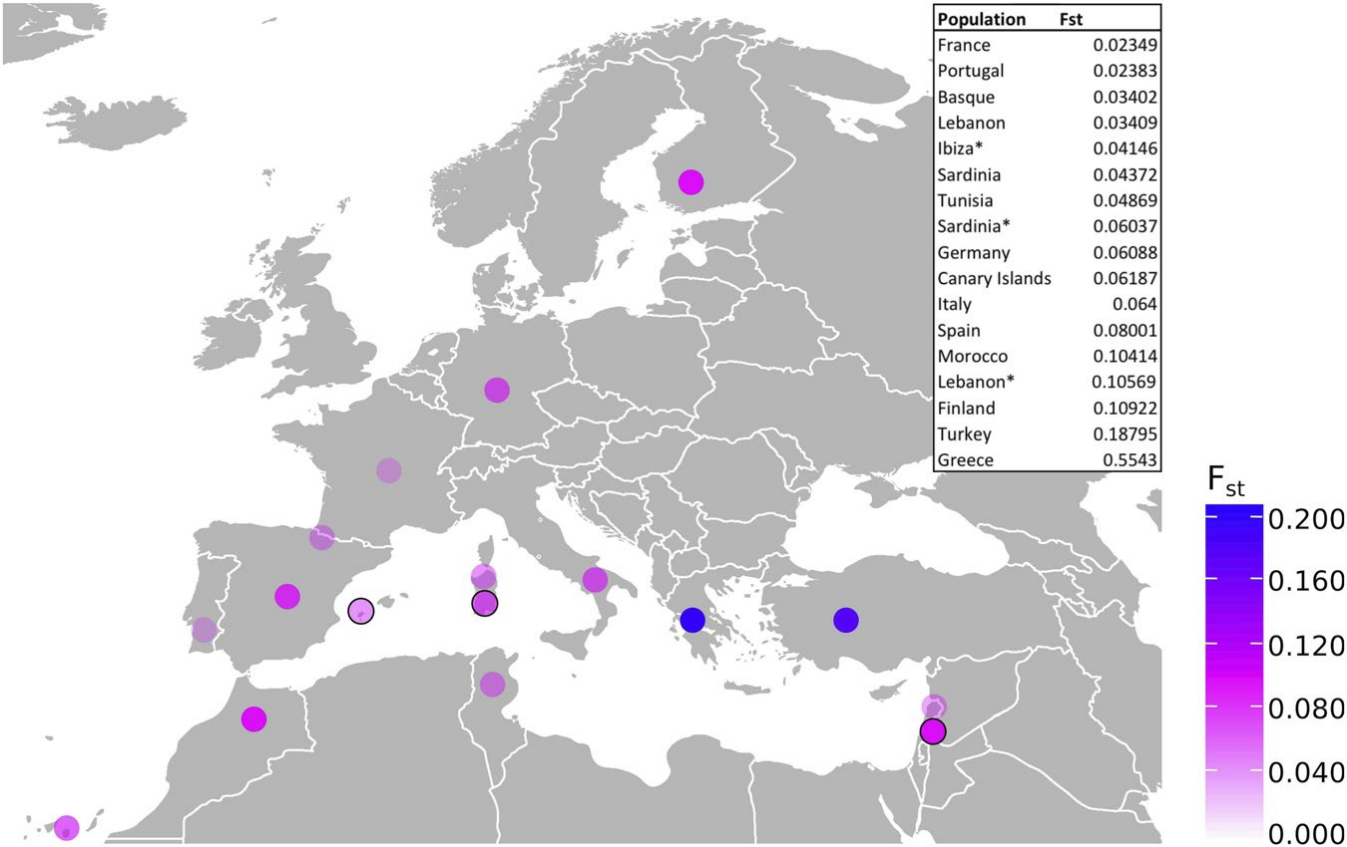
F_ST_ geographical mapping based on complete mitogenomes. Results of genetic distance (pairwise F_ST_) values between modern Ibizans and other modern Mediterranean, European and ancient Phoenician populations^3,34,41–47^. Dark blue values represent higher F_ST_ values (greater distance) and light pink represents lower F_ST_ values compared to modern Ibizans. Ancient Phoenician populations are identified by the black outline of the circles. Exact F_ST_ values are listed in the inset table. Ancient Phoenician populations are indicated in the table by asterisks.

Following the lack of DNA evidence for continuity between the Phoenician and modern Ibizan populations, we compared the modern and Phoenician mitochondrial data to other likely candidate source populations. Genetic distance (F_ST_) was calculated between modern Ibizans and multiple candidate source populations^34,41–47^. Pairwise F_ST_ between all populations were visualized in a MDS plot (Supplementary Data 3), to assess the genetic distance between Ibiza and candidate source populations in comparison to the genetic distance among source populations. The genetic distance between modern Ibizans and candidate source populations was then visualized on a map, to refine our estimates of populations maternally closely related to the modern Ibizans (Fig. 4).

**Figure 5:**
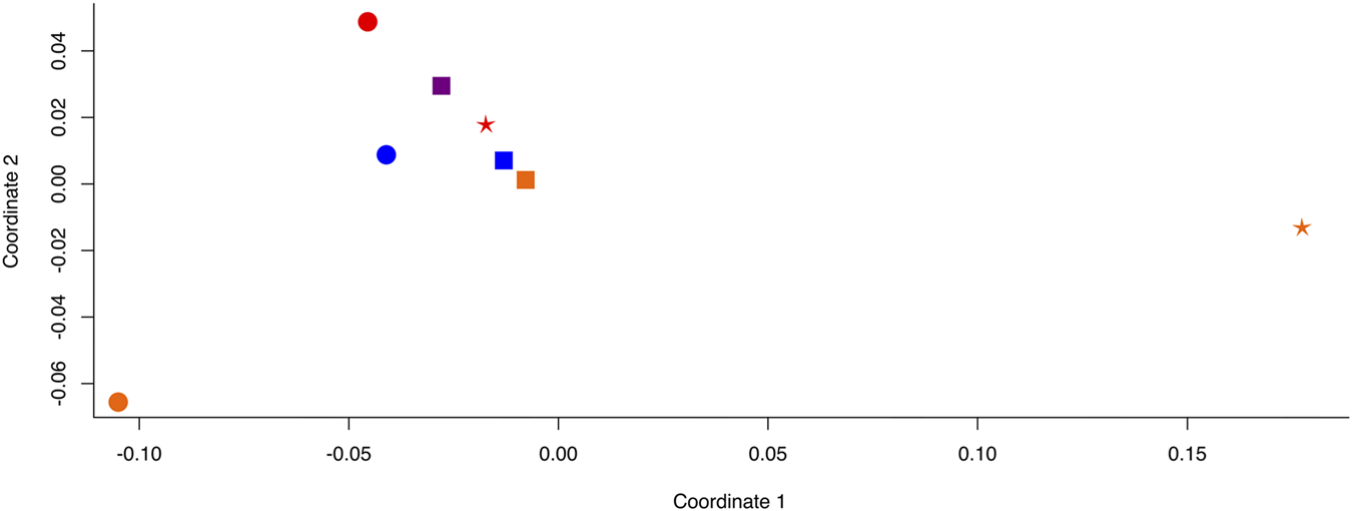
MDS of complete mitogenome sequence data, based on F_ST_: Ibiza (blue), Lebanon^3,29^ (orange), Sardinia^3,34^ (red), Spain^34,41,45^ (purple). Population datasets are grouped by age: pre-Phoenician (stars), Phoenician (circles) and modern (squares).

To determine if there were any patterns observed between Phoenician populations in the Mediterranean, we combined our ancient Phoenician data with previously published mitogenome data for a range of Mediterranean populations including pre-Phoenician, Phoenician and modern Sardinians^3,34^ and pre-Phoenician^29^, Phoenician and Modern Lebanese^3^ as well as modern mainland Spanish^34,41,45^ and performed multidimensional scaling of pairwise genetic distance (Fig. 5).

#### Analysis of whole genome shotgun data

**Figure 6:**
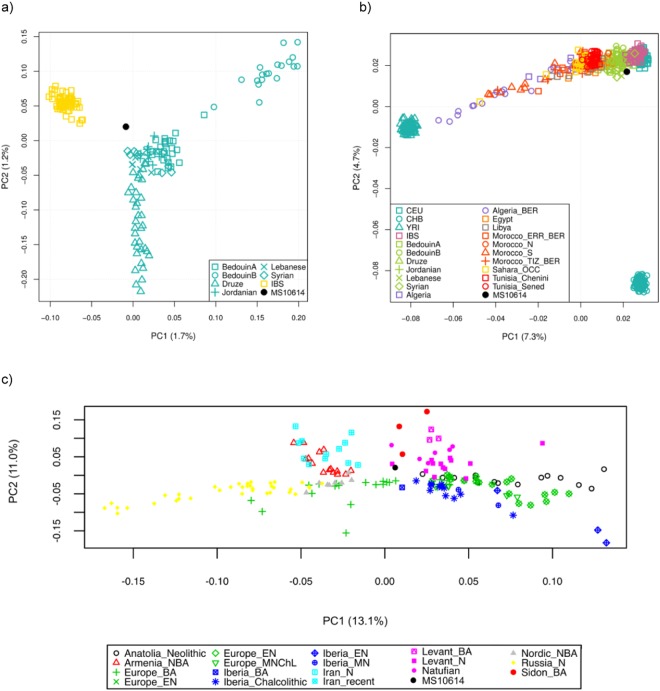
Principle Component Analysis of genome-wide SNP data for MS10614 with (**a**) modern Iberian and Levantine samples from the 1000 genomes dataset; (**b**) the same modern samples with the addition of North African populations from 1000 genomes; and (**c**) 311 ancient individuals from previously published SNP datasets^29,32^.

The origins of the Phoenician Ibizan individual (MS10614) were first assessed by constructing a PCA with the 1000 genome^33^ data from three worldwide populations, Asia (CHB), Europe (CEU) and Africa (YRI) in which MS10614 was located closest to the European samples (when including both tranversions only and transitions and transversions) (Supplemental Fig. 4a). To provide more context within the European cluster we first added to our initial PCA analyses data from modern Iberians^33^ (IBS) and Levantine populations from Jordan, Syria, Lebanon, and Israel^32^. The Ibizan sample plotted within the Levantine cluster, outside of the modern Iberians (Supplemental Fig. 4b). When considering more fine-grained analysis including only the Levantine and Iberian modern data with MS10614, the Ibizan positioned between the Iberian and Levantine samples, with more affinity to the Levantine populations (Fig. 6a). We conducted a further test, adding North African populations^48^ to the analyses (Fig. 6b) and see that MS10614 is situated more closely to the Levantine and Iberian samples and not the North Africans. Finally, since modern populations are likely to have changed significantly in the 2000 + years since the Phoenician expansion, we undertook similar analyses to assess the relationship between MS10614 and other ancient populations (Fig. 6c). The ancient sample MS10614 was also plotted with the modern Human Origins (HO) dataset for reference (Supplemental Fig. 4c).

**Figure 7:**
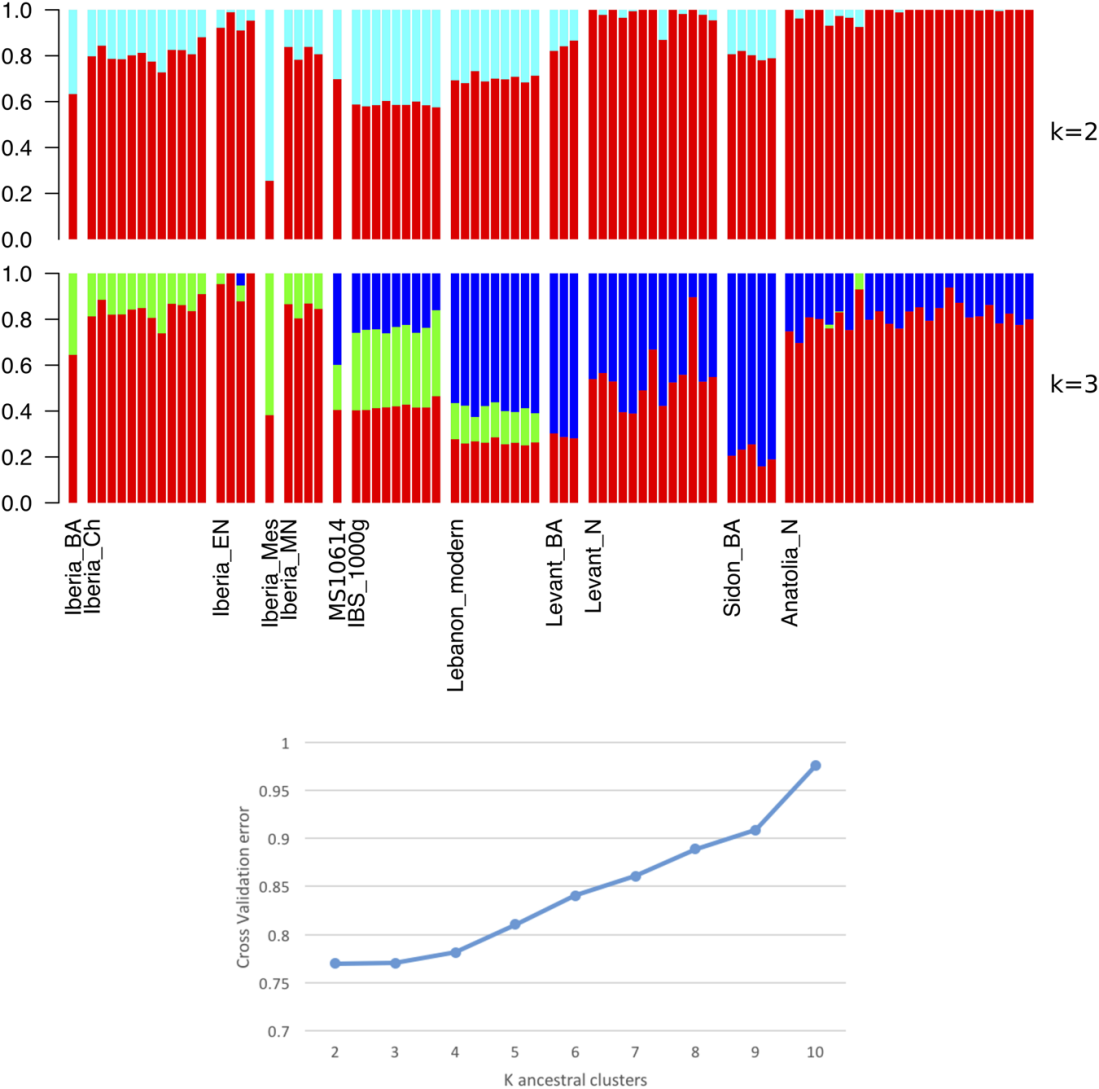
Subset of ADMIXTURE analysis results for sample MS10614 with ancient Iberian, Anatolian and Levantine^29,30^ and modern Spanish^33^, Tunisians^30^ and Lebanese^31^ whole genome datasets for k = 2 (cross-validation score = 0.76991) and k = 3 (cross-validation score = 0.77029). Cross-validation scores are displayed for ancestral clusters of K = 2 to 10.

The ancestry of MS10614 was also estimated using model-based clustering in ADMIXTURE^49^ (Supplementary Data 5). The most appropriate model was *k* = 2, determined by selecting the value of *k* with the lowest cross-validation error, though *k* = 3 had only a slightly higher cross-validation score, and both are shown in Fig. 7, which shows a subset of the data, consisting of the Iberian and Levantine populations. The model *k* = 3 shows a new component (dark blue) that impacts all of the Eastern Mediterranean populations and the modern Iberians. Sample MS10614 has an intermediate amount of this component, with more than modern Spanish, but less than modern Lebanese and ancient Levantines.

Outgroup f_3_-statistics were used to measure shared genetic drift between MS10614 and other ancient and modern populations. These results indicated that this Phoenician individual is most closely related to Neolithic aged samples from Iberia and Anatolia, plotting away from North African samples, providing further evidence for this individual having ancestry from both mainland Europe and the Near East (Supplementary Data 6). D-statistics were similarly supportive of gene flow between the Phoenician MS10614 and both Neolithic aged Iberian and Anatolian samples, however there was no statistical significance between the inferred gene flow from each of these regions with the Phoenician Ibiza individual.

## Discussion

Most Phoenician trading ports were initially founded in locations where there was already an established indigenous community, resulting in integration of locals into the growing Phoenician settlement. The settlement history of Ibiza offers a valuable opportunity to investigate the genetic makeup of a Phoenician population where there was a relatively very small indigenous population at the time of Phoenician arrival. Being a likely secondary Phoenician settlement from the city of Gadir, the early population of Ibiza also potentially provides information about Phoenician settlements on the Iberian mainland. The data presented here adds to our previous research investigating admixture in Phoenician settlements across the Western Mediterranean^3,23^. It also helps dissect and possibly identify the origins of the various populations that ultimately contributed to the genetic makeup of the modern Ibizans.

Our analyses of the mitogenomes of the ancient samples from Ibiza indicate a predominantly European maternal ancestry for the population (Fig. 1). Haplogroups H1 and H3, which either emerged from the Franco-Iberian refugia of the LGM^50,51^, or arrived with early Neolithic expansions, account for 50% of the Ibizan ancient samples (see Table 1). Szecseny-Nagy *et al*.^38^ also found similarly high rates of haplogroup H in their analyses of the Hyper-Variable Region (HVR) of Neolithic and Early Bronze Age Iberian samples. Investigating coding region SNPs, they showed that among haplogroup H, 65% belonged to hg H1 and 14% to hg H3. Particularly high frequencies of H1 and H3 are also seen in ancient and modern Sardinians^3,34^. The majority of the remaining haplogroups present in the Phoenician population of Ibiza, U4a, U5b, and T2b have all been identified in Mesolithic or Neolithic populations in Western Europe including Iberia^38,52,53^. Haplogroup U5b3 frequencies, though generally low across Europe, are the highest today in Sardinia and Spanish Andalusia^54^ and are thus also consistent with an Iberian origin or possible contacts with Phoenician Sardinia. Haplogroup J1c is thought to have an eastern Mediterranean origin and has been identified in several Neolithic samples in Eastern Europe and in a Chalcolithic sample (2880–2630 BCE) from the El Mirador Cave site, Atapuerca, Spain^55^. Haplogroup J1c has not been found previously in any ancient samples in the Levant and appears to have been distributed across Europe with the Neolithic expansion, suggesting that it may have been picked up in Northern Anatolia^30^. Olivieri *et al*.^34^ found a J1c3 haplotype in one of their ancient samples from Sardinia (MA74), dated to 6190–6000 calBP and thus its presence in our Puig des Molins samples is not unexpected, as this lineage was clearly present in the Western Mediterranean region at the time that Ibiza was settled. The primary Phoenician settlers of Ibiza are likely to have be males who sailed from Gadir and the presence of these haplogroups provides additional support to our previous work suggesting that local female integration was a hallmark of Phoenician settlement history across the Mediterranean basin^3^.

Our previous analyses of Phoenician mtDNA from Monte Sirai, Sardinia, another Western Mediterranean Phoenician and Punic settlement, indicated significant integration of indigenous women among the predominantly male Phoenician settlers^3^. We suggest a similar pattern is indicated for the Phoenician settlement of Ibiza. When we investigated the relationships of mitogenomes of Phoenician populations from Lebanon, Sardinia and Ibiza (Fig. 5) we found that there was no particular affinity amongst Phoenician populations (circles) when compared to pre-Phoenician or modern populations from those locations. This may indicate that local female integration was indeed a common strategy of Phoenician settlements. We expect that the integration of indigenous women from south central coastal Iberia into the Phoenician settlement of Gadir must have also occurred and that the ancient mitochondrial lineages observed Ibiza were brought along from Gadir or, alternatively, from some other Phoenician settlement in the Central or Western Mediterranean. Analyses of ancient Phoenician samples from Gadir and complete mitogenomic sequencing of ancient samples from across the Iberian Peninsula are needed to fully test this explanation. Analyses of HVR sequences from Neolithic and Early Bronze Age samples from the Iberian peninsula^38^ and modern southern Iberian populations^56^ indicate that there may be differences between the southwest and southeast Iberian populations, and it may be possible to locate particular regions of the Iberian coast from which these mtDNA lineages originated.

There was a sizable increase in the number of the Punic burials at Puig des Molins during the 5^th^ and 4^th^ centuries BCE, indicating a surge in the population size of Ibiza^10^. This increase is largely attributed by historians to the movement of people from other prospering Phoenician/Punic settlements in nearby Sardinia and Carthage^10^. Despite anthropological evidence supporting the presence of North and sub-Saharan African ancestry in Punic individuals from the Island of Ibiza^57^, mitochondrial lineages exclusively associated with North Africa or the Near East were not observed in our ancient Ibizan samples, although several of these lineages were previously identified in Phoenician samples from Sardinia, i.e. haplogroup N1b1a5^3^. Our results cannot, however, rule out the arrival of admixed individuals with European maternal ancestry from Phoenician sites in Sardinia, North Africa or the Iberian mainland. In fact, we previously identified a likely Iberian mtDNA haplotype, U5b2c1, in a young Carthaginian^23^, suggesting the presence of an admixed population there by late 6^th^ century BCE. We also note that haplogroups H1 and H3, while European in origin, are also found at high frequency in North African populations, particularly H1, which is today found at levels of 40–45% in northwest Africa^56,58^. Haplogroup H1bc was identified in a Bronze Age sample from Sidon^29^ and H1 is present in modern Lebanese^3^. It is therefore possible, that these H lineages could have been brought to Ibiza, with Phoenicians from either the Levantine homeland or North Africa. It is a more parsimonious suggestion, however, that their presence in Ibiza is due to a more direct European and probably and Iberian source, particularly given the lack of any mitogenomes of clear North African or Near Eastern origin identified in the ancient Ibizan population sampled thus far. The morphological characteristics indicating African ancestry in crania from Punic contexts in Ibiza identified by Márquez-Grant^57^ could be consistent with our aDNA results if the African admixture in Ibiza was male dominated. Alternatively, aDNA analyses of additional Phoenician samples from Ibiza may indeed find non-European mtDNA lineages, but to date, we have only observed what appears to be a typical European, and primarily Western European signature.

Unfortunately, to really dissect population mtDNA histories generally, and those of southwest Europe or any region with high rates of haplogroup H in particular, complete mitogenome sequencing is necessary. Despite the large sample sizes for studies of HVR sequences for the Iberian Peninsula^38,59^, there are few complete ancient mitogenome sequences publicly available particularly beyond the Basque region. As Hernandez *et al*.^56^ point out, secure assignment to haplogroup H is not possible based on control region sequencing alone, as it requires identification of T7028C. Further sub-typing of haplogroup H also requires the identification of key coding region SNPs (for example, G3010A for H1, T6776C for H3).

In addition to the ancient Ibiza samples, we sequenced a complete mitogenome of a Late Chalcolithic/Early-Bronze Age sample (MS10589) from the Ca na Costa site on the island of Formentera which was determined to belong to the K1a1b haplogroup. Mathieson *et al*.^55^ identified K1a1b1 in a Middle Neolithic Iberian sample (Mina3) with an archaeological context dated to 3900–3600 BCE. In their analyses of mtDNA hypervariable region sequences obtained from ancient samples from Neolithic and Iron-Age necropoli from Mallorca and Menorca, Simón *et al*.^39^ found that 4.34% belonged to Haplogroup K, with the highest frequency (10%) in the oldest (Neolithic) necropolis, Cova des Pas, on Minorca. Our K1a1b result may indicate that the initial settlement of Formentera, prior to the arrival of the Phoenicians, also likely came from the mainland or via Mallorca, an observation presented by Bellard^4^. Olivieri *et al*.^34^ found K1a and K1b1b1 in pre-Phoenician Sardinians, but we have yet to find haplogroup K in any of our Phoenician samples.

Ibiza has had a complex history of resettlement periods since the time of the Phoenicians. In addition to a Phoenician founding population, modern Ibizans would have had potential genetic influences from later Punic settlements, like Carthage and our ancient DNA data are consistent with this scenario. Historic records would suggest that the Ibizan genetic signature was also likely influenced by the arrival of Arab and Berber populations during the first millennium CE and finally, from 1200 CE, it was said to be overwhelmed by mainland colonists. This might explain the discontinuity between the ancient and modern DNA results we observed, shown in Fig. 3 and is consistent with the results of Ramon and colleagues^13^, based on HLA markers and blood groups in Ibizans, and earlier mtDNA^14^ and Y-chromosome^15^ studies which show affinities to North African and Near Eastern populations.

Our TempNet results showed little continuity between the Phoenician inhabitants of the island and the modern Ibizan population. No shared haplotypes were found and only one haplogroup, T2b, was found in both groups. Perhaps most surprising was that neither haplogroups H1 or H3 were found in the modern Ibizans. These two haplogroups were not only the most common in our ancient population but are found at high frequencies in both modern North African and Western European populations generally^56^. The modern Ibizan population is singularly unusual in the entire Western Mediterranean region for their apparent lack of these two common haplogroups. It is possible that the mtDNA frequencies are so different from the ancient samples due to random genetic drift, which is common in small island populations. The small sample size (n = 18) could also be a factor, though our result indicating that the modern Ibizans are genetically quite different to mainland populations is consistent with previous studies^13,14,16,39^.

Haplogroups T1 and V, found in the modern Ibizans, are lineages found at reasonably high frequencies in the Near East. Haplogroup V is also found in both ancient and modern populations from the Iberian Peninsula and in ancient samples from Mallorca and Minorca, and T1 is also found at low frequency in mainland Spain today^14,32,55^. These lineages are also found in Berbers which could explain their presence in the modern Ibizans. Haplogroup L2c however, which was identified in two unrelated, modern Ibizans, has not been found in Berbers^60^. It is typically a West African, sub-Saharan lineage and it may have been introduced during the Islamic expansion which had significant exchange with the entire African continent. Alternatively, it could have been a more recent arrival, perhaps the result of the transatlantic slave trade^59^. Botigué *et al*.^61^, showed in their whole genome analyses that sub-Saharan ancestry was less than 1% in Europe, except for the Canary Islands, where Maca-Meyer *et al*.^62^ found L2c in both 17^th^ and 18^th^ century historic and modern individuals. Another sub-Saharan lineage, L1b has also been identified in a Late Chalcolithic population in central Iberia^38^ and in one 7^th^ century CE sample from Mallorca^39^.

The remaining lineages identified in modern Ibizans are all either exclusively European markers (e.g. J2b1a^63^) or are widely distributed across Europe and the Near East and identifying the specific origin of the population influx that causes the discontinuity between ancient and modern Ibizans is difficult. The F_ST_ results for the complete mitogenomes shown in Fig. 4 do not indicate a single likely source, though the closest population to modern Ibizans appears to be France. France may be acting as a proxy for a more likely source of settlers to Ibiza, namely Catalonia, since it has been shown to be genetically closely related^64^. Further whole genome analyses of the modern indigenous Ibizans would provide the necessary data to fully assess the origins of this population, but from a mitochondrial perspective, it appears that they are not directly related to the Phoenician and Punic founding populations and thus this is not the explanation for the unusual genetic signature.

While the mitochondrial evidence suggests that the founding female population of Ibiza was primarily derived from a mainland Iberian source, the results of the whole genome analyses of sample MS10614 indicate a significant Eastern Mediterranean/Levantine component. Sample MS10614 comes from an individual that was part of a collective burial inside a Punic hypogeum at Can Portes des Jurat, Cas Molí^20^. The sample was directly radiocarbon dated to 361–178 cal BCE. The archaeological context of the burial also suggests a 3^rd^ century BCE date and thus this individual lived during the period of greater Punic influence of Ibiza or just after this period of population growth.

As can be seen in Fig. 6a,b, the Ibizan Phoenician sample, MS10614, plots in between modern Levantine and Iberian populations but closer to both of these than to modern North African populations. In Fig. 6c, it is positioned most closely to a Levantine Neolithic sample, but in between a Sidon Bronze Age and European/Iberian Bronze Age samples. The ADMIXTURE result (k = 2) in Fig. 7 also indicates that the genetic ancestry of this individual was intermediate between an Iberian Bronze Age and Levantine Bronze Age individuals. The result of k = 3, however, shows a third component (dark blue) found in Anatolian and Levantine Neolithic and Bronze Age individuals. This component is present in the ancient Ibizan and also present in modern Spanish. We suggest that this third component represents the Western Iranian Neolithic farmers that had admixed with the Levantine and Anatolian Neolithic populations^30^. Levantine Bronze Age populations were further admixed with this component from both Anatolian Chalcolithic and Levantine Neolithic ancestry. This component is not present in ancient Iberians, supporting the notion that the main ancestral population of early European farmers were the Neolithic North-Western Anatolians before they were admixed with the Iranians^30^. The presence of this component in modern Iberians is likely the result of historic influences.

The mtDNA haplogroup of this individual, T2b, indicates that the direct maternal ancestry is likely local and the Levantine contribution may therefore represent paternal ancestry. This finding is in line with previous work by our group using Y-chromosome data showing East Mediterranean Y signatures across the Phoenician settlements in the West, including Ibiza. These signatures, attributed to the Phoenicians, were found to be present at the frequency of 6% among the modern male populations studied, indicating a substantial Phoenician male presence in settlements across the Western Mediterranean^65^. The Near Eastern component to the genome of this individual may represent that of the founding Phoenician population, though the possibility that this individual or a recent ancestor may have arrived directly from the Levantine homeland or from Punic settlements in North Africa or elsewhere in the Phoenician interaction network cannot be ruled out, though the sample does not appear to be closely related to modern North African populations as shown in Fig. 6b.

In conclusion, our results show a complex pattern of settlement of the island of Ibiza. We demonstrate a clear genetic discontinuity between the early Phoenician settlers and the modern inhabitants of the island based on the mtDNA results. Thus, the unusual genetic signature of modern Ibizans is not likely to be the result of their Phoenician ancestry, at least from a mitochondrial perspective. It appears that multiple population arrivals through invasions or other movements combined with periods of population instability since the early Phoenician settlement may have led to a total reshuffling of the genetic makeup of this island. Over the last several centuries Ibiza witnessed population growth supported by the arrival of mainland populations to the island, followed by significant population reduction resulting from the bubonic plague and malaria^13^. These events, combined with a founder effect, and inbreeding common in islands with a relatively small population such as Ibiza, could have resulted in the loss of the indigenous mtDNA signatures observed in the ancient samples we analysed. It appears that the lineages present in the Phoenicians of Ibiza were replaced by different European mtDNA haplogroups (which appear to be most closely related to those of modern French but likely also similar to Catalonians). Despite this lack of continuity observed in the mitochondrial genomes, previous Y chromosome analyses suggest that there is still some Phoenician signature in the modern Ibizan population. This is consistent with historical evidence suggesting that Phoenician influence in the West was male dominated and indicates that there was not a total replacement of the Ibizan founding population. Comparisons of the ancient Phoenician whole genome data with whole genome data from modern Ibizans will help clarify this point further. Finally, the whole genome data obtained from the ancient Ibizan sample belonged to an individual with a significant Eastern Mediterranean component, suggesting an admixed Phoenician community in Ibiza during the 3rd century BCE. While this result is consistent with the archaeological evidence and further indicates that diversity was a hallmark of Phoenician societies, it also highlights the complexity of island population settlements and underscores the importance of the inclusion of ancient DNA analysis in population genetics.

## Methods

### Archaeological sites and samples

Permits and approval for this study were obtained from the office of the Director General of Antiquities in Lebanon (permit number 4290, 6 November 2015) for all Lebanese samples and from the Museu Arqueològic d’Eivissa i Formentera for the Spanish samples and all methods were performed in accordance with the relevant guidelines and regulations.

Puig des Molins (windmill Mountain) is one of the largest necropoli discovered to date in west Phoenicia. Used since the 7^th^ century BCE, it spans an area around 50,000 m^2^, with close to 3000 tombs. At least three burial rituals can be distinguished in Puig Des Molins. The first between the 7^th^ and 6^th^ centuries BCE used cremation. The second coincided with significant population growth on the island during the 5^th^ and 4^th^ centuries BCE and corresponded to inhumation using the spectacular hypogea cut in the rock. The third, between the 3^rd^ and 2^nd^ centuries BCE witnessed the brief return of cremation. This necropolis has yielded many of the most important clues and indices that led to the understanding of the funerary rituals of the early Phoenician settlers of Ibiza.

Cas Molí is a Punic hypogeum situated near the Bay of Saint Antoni the Portmany, in the Western coast of the Island. Inside were recovered the bone remains of at least 15 individuals. The pottery found allow to situate the chronology of the site in 3^rd^–2^nd^ centuries B.C.

Ca na Costa is a megalithic funerary site on the island of Formentera and dating from the Late Chalcolithic to early Bronze age (2000–1600 BCE). The site was excavated between 1974 and 1977 and the remains of 8 individuals along with associated funerary  items were recovered in the circular stone tomb.

### Modern DNA samples

A total of 18 DNA samples were obtained from Ibizan individuals first sampled by researchers from the Institut de Biologia Evolutiva (CSIC-UPF), Barcelona, as part of a study on Y chromosomes and surnames. Previously, data on Y-chromosome SNPs and STRs in four of those samples had been part of a wider study on surnames and the Y chromosome^66^. Saliva samples were collected with full written informed consent after revision and approval by the Institutional Review Board of the Comitè Ètic d’Investigació Clínica-Institut Municipal d’Assistència Sanitària (CEIC-IMAS) in Barcelona (2016/6723/I) and all methods were performed in accordance with the relevant guidelines and regulations. Modern DNA samples were amplified in two long range PCR products, produced using primers HUM-LR1 and HUM-LR2 described previously^24^. The PCR products were pooled and 1 µg of the products, in equal molar concentrations, were mechanically sheared using sonication to produce fragments of approximately 500 bp in length. Blunt end repair, ligation of sequencing adaptors, sample barcoding and pooling were carried out following Meyer and Kircher^67^ and Kircher and Kelso^68^ with modifications for Illumina sequencing adaptors. Pooled samples were sequenced on one lane of the Illumina MiSeq in a 2 × 250 base paired-end run with version 2 chemistry at the Otago Genomics and Bioinformatics Facility (OGBF).

#### Ancient DNA extraction and library preparation

All aDNA extraction and library preparation (until PCR) was carried out in a purpose built aDNA facility at the University of Otago^69^. DNA was extracted from 13 teeth recovered from two archaeological sites on Ibiza and one site on Formentera. All teeth were rinsed in 5% bleach, followed by washing with distilled water for multiple washes to remove any bleach residues and drying overnight. DNA was extracted from ~150 mg of ground tooth, following a silica-based extraction protocol^70^. One extraction negative was prepared alongside every five extracts and processed alongside extracts for the remainder of the lab work.

#### Mitochondrial DNA enrichment and sequencing

For the mitochondrial genome sequencing, libraries were prepared and hybridisation capture used to target the human mitochondrial genome according to the protocol described by Matisoo-Smith *et al*.^23^. Mitochondrial genome libraries were pooled and sequenced using a 2 × 75 bp paired-end sequencing kit on an Illumina MiSeq platform at the Otago Genomics and Bioinformatics Facility (OGBF).

#### Mitochondrial DNA sequence processing and sequence alignment

Resulting sequencing data from the ancient mitochondrial libraries were processed according to methods described previously^3^. Complete mitogenome sequences were subsequently aligned with one another and previously published mitochondrial sequences using mafft^71^.

#### Authentication and contamination assessment

Throughout the library preparation process, ‘negative’ libraries were handled alongside sequencing libraries and visually checked by gel electrophoresis and on the BioAnalyser to ensure that these did not contain DNA. MapDamage^72^ was used during processing of sequencing data to check nucleotide mis-incorporation patterns and fragment length distribution of sequencing libraries, as an indication that the resulting data looked degraded (Supplementary Data 2). During this step, the –rescale option in mapDamage was applied to rescale the quality scores of likely damaged bases towards the ends of reads. Mitochondrial DNA contamination was estimated using contamMix on the basis of a majority rule mtDNA consensus sequence and an alignment of 311 worldwide mtDNA sequences^73^ (contamMix data shown in Table 1).

Due to the relatively high contamination rate (14%) of sample MS10589, we used PMDtools with a damage threshold of 3 to select only those reads that were damaged^74^, resulting in 65.6% coverage (average coverage of 1.3X). When the VCF was loaded into HaploGrep we obtained the haplogroup call of K1a1b (which is consistent with the original call of K1a1b1) and confirmed 7 informative SNPs, ranging from between 2 and 4X coverage, thus we included this sample in further mitogenome analyses.

#### MtDNA sequence analysis

Network Analyses – mitogenome and HVR: In order to identify relationships between the modern and Phoenician Ibizans and potential source populations, aligned mitochondrial sequences (complete mitogenome and partial HVR) were used to construct median joining networks in PopArt^75^. A full list of populations included in each network is available in the Supplementary Tables 3 and 4.

Temp net: The modern and ancient mitogenome sequences generated in this study were aligned and used to create a TempNet temporal haplotype network^40^ to assess whether the modern Ibizan population were directly descended from the Phoenician Ibizan population.

MDS: The genetic distance between modern Ibizans and candidate source populations, based on complete mitogenomes, were compared using a multi-dimensional scaling analysis. A MDS plot was also generated to compare mitogenome sequences from modern and Phoenician Ibizan individuals to modern, Phoenician and pre-Phoenician (depending on data availability) populations from the Spain, Sardinia, Lebanon and Egypt using a multi-dimensional scaling analysis. The genetic distances between populations were calculated using DnaSP v6^76^ and F_ST_ values were visualised in a two-dimensional MDS plot, using the pcoa function in the ‘ape’ package for R^77^.

#### Shotgun sequencing of whole genome

One individual, MS10614, was selected for shotgun sequencing based on the high coverage of the mitochondrial genome sequencing results. The amplified sequencing library prepared for hybridisation capture was submitted for shotgun sequencing using a 2 × 75 bp paired-end sequencing kit on one lane of a HiSeq at the Otago Genomics and Bioinformatics Facility. Sequencing reads were mapped to a human reference sequence (UCSC hg19), and genotypes were called by sampling a random read per SNP in the Human Origins SNP panel, using the ‘pileupCaller’ tool (https://github.com/stschiff/sequenceTools/tree/master/src-pileupCaller). The resulting genotypes were merged with ancient DNA datasets from Lazaridis *et al*.^30^ and Haber *et al*.^29^ and modern DNA datasets of Iberians (IBS) from the 1000 Genomes Project Consortium^33^, Tunisians from Lazaridis *et al*.^32^ and Lebanese from Haber *et al*.^31^.

#### Analysis of whole genome data

Sex determination: To determine the genetic sex of the sample MS10614, sequenced using shotgun sequencing, we compared the number of Y chromosome alignments (3516) to the total number of X and Y chromosome alignments (827,587) as described by Skoglund and colleagues^27^. The sample was determined to be ‘male’ if the ratio of Y and autosomal coverage was greater than 0.25. Our result of 0.0042 would indicate the sex was female, though we suggest the low coverage overall could contribute to this result which contradicts the morphological characteristics that were recorded by the archaeologist at the time of excavation (Supplemental Data 1).

PCA: When we projected MS10614 into the context of the three worldwide modern populations (Supplemental Data 4 and Fig. 6a,b), we ran the analysis twice: one taking into account the transversions only, and one including even the transitions. This approach allowed us to see if the same pattern was reproduced or not and, since in both cases the behaviour observed resulted to be the same, we decided to stand with the more inclusive file (57,221 variants).

In all the considered scenarios involving modern populations and MS10614, the Principal Component Analyses were produced calculating the eigenvectors with smartpca; the ancient sample was projected setting the lsqproject parameter to yes in the smartpca parameter file. In every case we only took into account those SNPs that were common to the merged datasets and the ancient sample. No minor allele frequency filter, or LD pruning were performed. In modern samples, only variants and samples with many missing calls were filtered out (>5% and >10%, respectively). Also, variants failing a Hardy-Weinberg equilibrium exact test were discarded (p < 10^−5^).

For the ancient samples, PCA was performed using the merged dataset containing the Phoenician Ibizan (MS10614), ancient individuals from Lazaridis *et al*.^30^, and Bronze Age samples from Sidon^29^, using the ‘smartpca’ software from the Eigensoft package^78^.

Admixture: The ADMIXTURE v 1.3 software^49^ was used on a subset of the above merged dataset, comprised of the Phoenician Ibizan, Neolithic Anatolians, Late Neolithic-Bronze Age Europeans, Bronze Age Iberians, Bronze Age Levantines, Neolithic Levantines, modern Lebanese, modern Spanish, Bronze Age Sidon samples, and modern Tunisian populations. The ancestry proportions of these populations were assessed by testing clusters of *K* = 1–10 (shown in Supplemental Data 5).

F_3_ and D statistics: Outgroup f_3_ statistics were computed using the ‘qp3pop’ tool from the Admixtools package^79^ in the form of f_3_(Mbuti; X; MS10614) where ‘X’ tests all populations in the merged dataset. Based on the results of the outgroup f_3_ statistics, the ‘qpDstat’ tool from the Admixtools package^50^ in the form of *D*(MS10614; X; Y; Mbuti), where ‘X’ and ‘Y’ tests populations identified as being ancestral to MS10614 in the f_3_ and PCA analyses (Supplemental Data 6 and Table 5).

## Electronic supplementary material

Supplementary Tables

Supplementary Information

## Data Availability

Modern and ancient mitogenomes presented in this paper are available under the accession numbers MH43585-43559. We are currently in the process of submitting the mapped BAM files for the mitochondrial samples and the shotgun sequence data to the NCBI short read archive.
