# Supplementary material for: Ancient DNA of Phoenician remains indicates discontinuity in the settlement history of Ibiza
Source: Sci Rep. 2018 Dec 4;8:17567. doi: 10.1038/s41598-018-35667-y (PMC6279797; doi:10.1038/s41598-018-35667-y)
Supplement: Supplementary file 2 — Supplementary Information [file 41598_2018_35667_MOESM2_ESM.docx]

**Supplementary Information**

Title: Ancient DNA of Phoenician remains indicates discontinuity in the settlement history of Ibiza

Pierre Zalloua^1§^*, Catherine J. Collins^§2^, Anna Gosling^2^, Simone Andrea Biagini^3^, Benjamí Costa^4^, Olga Kardailsky^2^, Lorenzo Nigro^5^, Wissam Khalil^6^, Francesc Calafell^3^ and Elizabeth Matisoo-Smith^2^*

Affiliations:

1. School of Medicine, Lebanese American University, Byblos, Lebanon
2. Department of Anatomy, University of Otago, PO Box 56, Dunedin 9054, New Zealand.
3. Department de Ciències Experimentals i de la Salut, Institute of Evolutionary Biology (CSIC-UPF), Universitat Pompeu Fabra, Barcelona, Spain.
4. Museu Arqueològic d'Eivissa i Formentera, Universitat de Barcelona, Illes Balears, Spain
5. Facoltà di Lettere e Filosofia, Università di Roma, La Sapienza, Rome Italy.
6. Department of Arts and Archaeology, Lebanese University, Lebanon

^§^ Equal Contribution

*Corresponding Authors. Requests for materials should be addressed to PZ ([pierre.zalloua@lau.edu.lb](mailto:pierre.zalloua@lau.edu.lb) ) or EM-S ([matisoo-smith@otago.ac.nz](mailto:matisoo-smith@otago.ac.nz) )

**Supplementary Data 1**

Nine complete mitogenomes were successfully obtained from 13 ancient samples provided by B. Costa of the Museu Arqueologic d'Elvissa i Formentera, Ibiza. The archaeological context of the samples successfully sequenced are below:

MS10589 - Tooth - Ca Na Costa Sector 3 (excavated in 1975) Island of Formentera (Balearic Islands), Spain. This sample comes from a megalithic chamber tomb dated between 2000-1600 BC, this is Final Chalcolithic/Early Bronze Age.

MS10612 - Tooth - Calle Leon 10-12 Estrato II (excavated in 1983-84) at Necropolis of Puig de Molins. Detailed location: plot at Leon Street, number 10-12. Ibiza Town, Island of Ibiza (Balearic Islands). The sample comes from a set of disordered human remains in Stratum II, dated to the 3^rd^ to 2^nd^ century BCE.

MS10613 - Tooth - Calle Leon 10-12 Hip 3 BOCA (excavated in 1983-84) at Necropolis of Puig de Molins. Detailed location: plot at Leon Street, number 10-12. Ibiza Town, Island of Ibiza (Balearic Islands). This sample comes from human remains from the shaft of Hypogeum 3. All the human remains inside the chamber had been disordered (possibly during the Late Punic Period) but can be dated to the 4th century BCE.

MS10614 - Tooth - Can Portes d'es Jurat hipogeo ca's moli (excavated in 1973) at Ca's Molí. Detailed location: Can Portes d’es Jurat. Municipality of Sant Antoni de Portmany, Island of Ibiza (Balearic Islands). The sample (probable male) comes from a collective burial inside a Punic hypogeum at Can Portes des Jurat. The chronology of the burial is 3rd century BCE.

MS10616 - Tooth - Puig des Molins, INH 5 (excavated in 2000) at Puig des Molins, Detailed location: NW Sector. Ibiza Town, Island of Ibiza (Balearic Islands). This sample comes from an inhumation burial of an adult located at the top of a shaft hewn in the bedrock (Inhumation 5/2000). Its estimated date is early Roman.

MS10617 - Tooth - Puig des Molins, INH 6 (excavated in 2000) at Puig des Molins, Detailed location: NW Sector. Ibiza Town, Island of Ibiza (Balearic Islands). This sample comes from a child burial in a pit grave excavated in the ground (inhumation 6/2000). Its estimated age is 5th-4th centuries BCE.

MS10619 - Tooth - Puig des Molins, INH 5 (excavated in 2005, rescue) at Puig des Molins. Detailed location: Subsoil of Monographic Museum Sector, Ibiza Town, Island of Ibiza (Balearic Islands). The sample comes from an adult individual buried inside a sandstone sarcophagus in a pit grave (inhumation 5/2005). The estimated chronology of the burial is 5th-4th centuries BCE.

MS10620 -Tooth - Puig des Molins, INH 7 (excavated in 2005) at Puig des Molins. Detailed location: Subsoil of Monographic Museum, Ibiza Town, Island of Ibiza (Balearic Islands). This sample comes from a child buried inside an amphora, and this is in a pit grave excavated in the ground (Inhumation 7/2005). The estimated chronology of the burial is 4th century BCE.

MS10622 - Tooth - Puig des Molins, INH 15 (excavated in 2005) at Puig des Molins. Detailed information: Subsoil of Monographic Museum, Ibiza Town, Island of Ibiza (Balearic Islands). This sample comes from an individual buried in a grave pit (inhumation 15/2005). The estimated chronology of the burial is 4th century BCE.

**Supplementary Data 2**


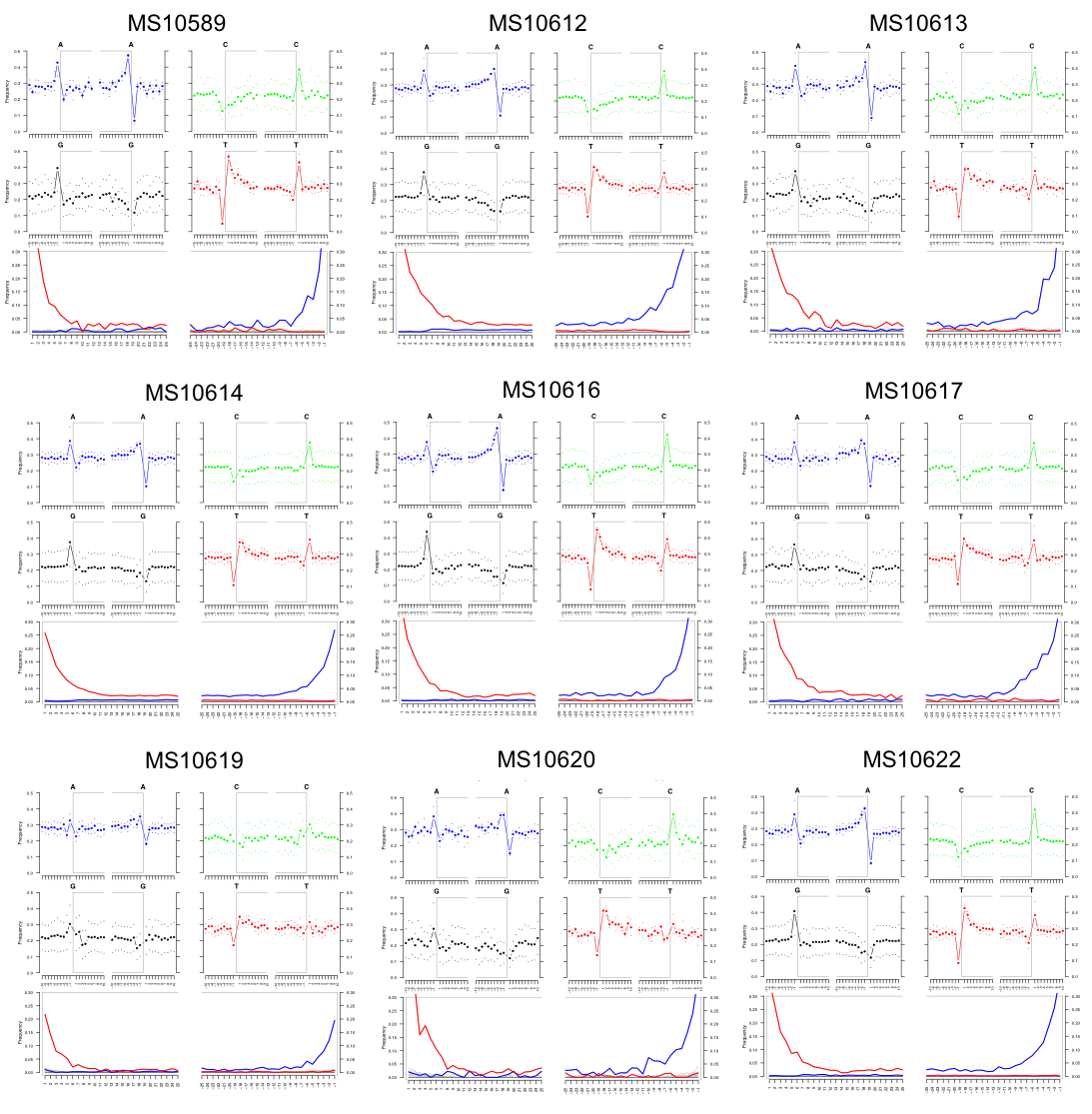


**Supplementary Figure 2a** Ancient DNA damage patterns for nine ancient (mitochondrial capture) samples from Ibiza.


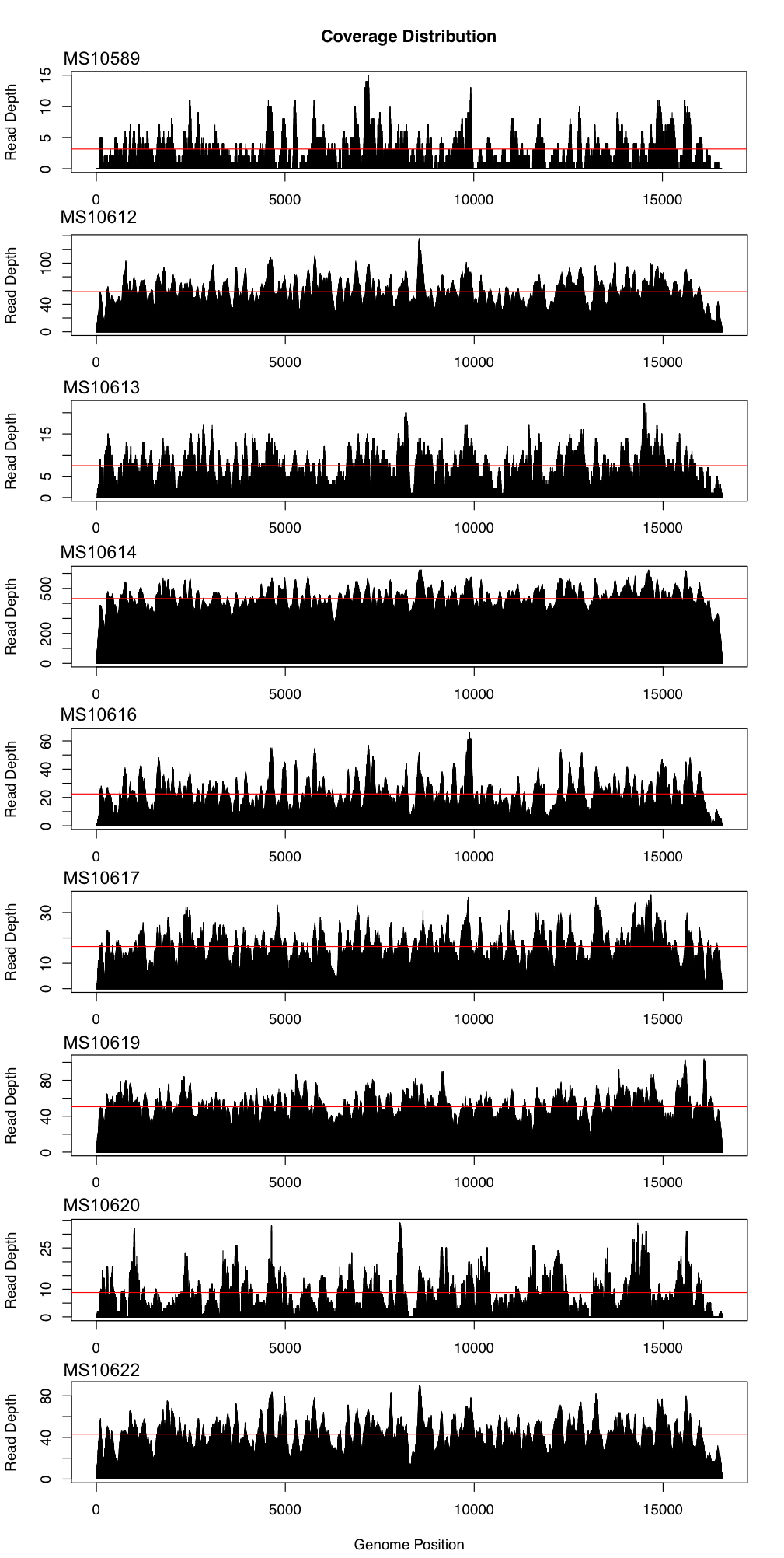


**Supplementary Figure 2b** Read depth across the mitochondrial genome for the nine ancient (mitochondrial capture) samples from Ibiza. Average read depth is indicated by the red line.


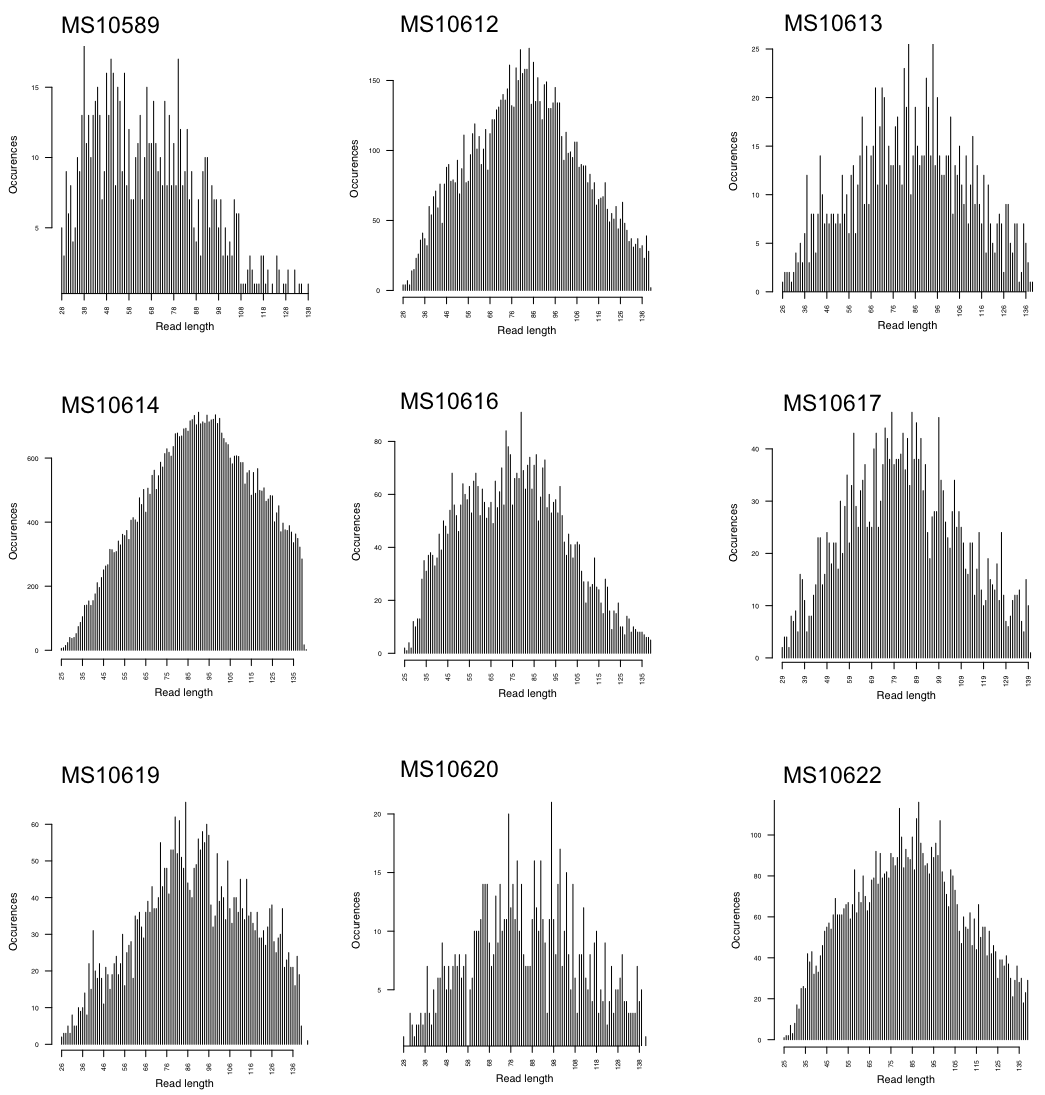


**Supplementary Figure 2c** Merged fragment length distribution of nine ancient (mitochondrial capture) samples from Ibiza.

**Supplementary Data 3**


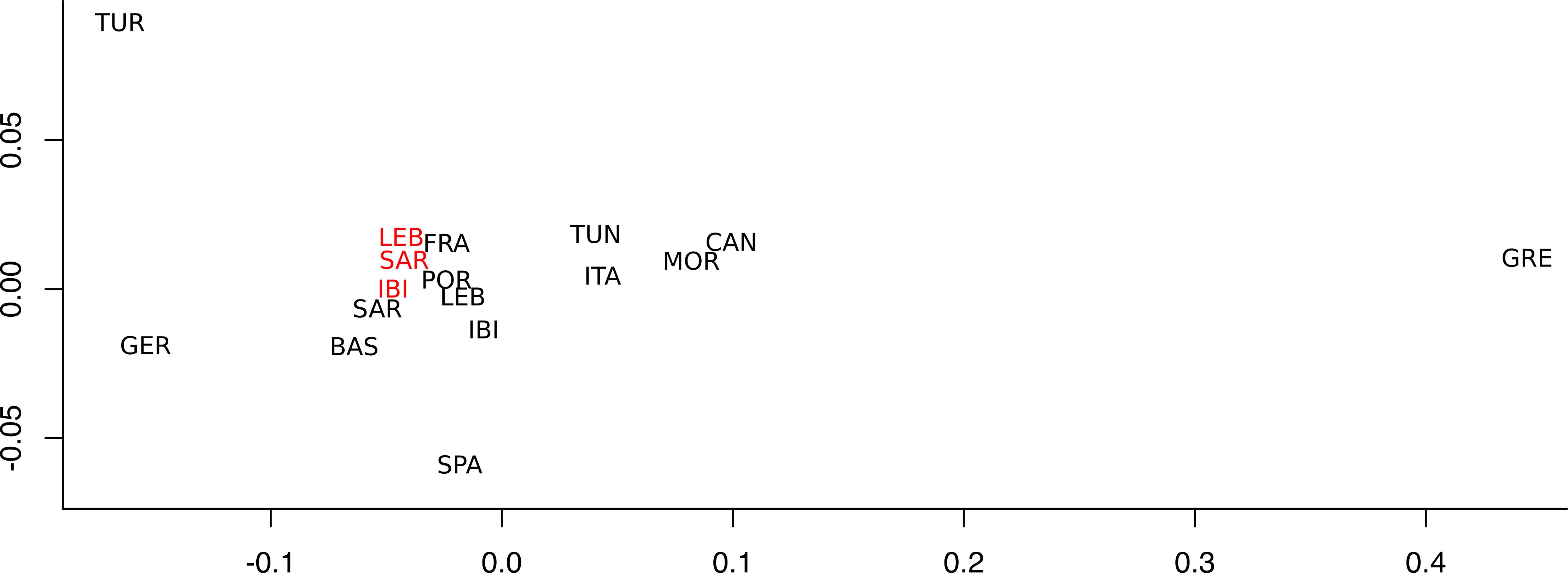


**Supplementary Figure 3a**. MDS of complete mitogenome sequence data from candidate source populations for modern Ibizans, based on genetic distance (pairwise F_st_). Modern populations are indicated by black text and Phoenician populations are indicated by red text; (LEB:Lebanon,SAR:Sardinia,FRA:France,IBI:Ibiza,MOR:Morroco,ITA:Italy,SPA:Spain,

BAS:Basque,POR:Portugal,GER:Germany,TUN:Tunisia,GRE:Greece,CAN:Canary Islands, TUR: Turkey)

|  | **Basque** | **Lebanon** | **Lebanon** | **Ibiza** | **Sardinia** | **Germany** | **Canary** | **Italy** | **France** | **Greece** | **Tunisia** | **Turkey** | **Spain** | **Ibiza** | **Morocco** | **Sardinia** | **Portugal** |
| --- | --- | --- | --- | --- | --- | --- | --- | --- | --- | --- | --- | --- | --- | --- | --- | --- | --- |
| **Basque** | - |  |  |  |  |  |  |  |  |  |  |  |  |  |  |  |  |
| **Lebanon** | 0.02157 | - |  |  |  |  |  |  |  |  |  |  |  |  |  |  |  |
| **Lebanon** | 0.01842 | 0.01589 | - |  |  |  |  |  |  |  |  |  |  |  |  |  |  |
| **Ibiza** | 0.0034 | 0.00814 | -0.03665 | - |  |  |  |  |  |  |  |  |  |  |  |  |  |
| **Sardinia** | 0.02101 | 0.00895 | -0.0168 | -0.01323 | - |  |  |  |  |  |  |  |  |  |  |  |  |
| **Germany** | 0.02914 | 0.02455 | -0.01626 | -0.05195 | -0.04125 | - |  |  |  |  |  |  |  |  |  |  |  |
| **Canary** | 0.02726 | 0.00616 | 0.03984 | 0.00977 | 0.03167 | 0.03425 | - |  |  |  |  |  |  |  |  |  |  |
| **Italy** | 0.04103 | 0.03226 | 0.04479 | 0.03103 | 0.05077 | 0.08027 | -0.00988 | - |  |  |  |  |  |  |  |  |  |
| **France** | -0.01624 | -0.00121 | -0.03976 | -0.0453 | -0.00375 | -0.0354 | 0 | -0.00633 | - |  |  |  |  |  |  |  |  |
| **Greece** | 0.49613 | 0.43818 | 0.45887 | 0.47162 | 0.46229 | 0.6 | 0.21667 | 0.33858 | 0.4507 | - |  |  |  |  |  |  |  |
| **Tunisia** | 0.04716 | 0.0319 | 0.04359 | 0.0363 | 0.04227 | 0.0616 | 0.00976 | 0.03788 | 0.0272 | 0.33869 | - |  |  |  |  |  |  |
| **Turkey** | 0.11922 | 0.11106 | 0.05517 | 0.08776 | 0.07996 | 0.08696 | 0.15254 | 0.14402 | 0.05839 | 0.61111 | 0.12459 | - |  |  |  |  |  |
| **Spain** | 0.03747 | 0.02764 | 0.0612 | 0.02976 | 0.0561 | 0.09359 | 0.00171 | 0.01681 | -0.00104 | 0.44418 | 0.04498 | 0.19046 | - |  |  |  |  |
| **Ibiza** | 0.04532 | 0.04961 | 0.01871 | 0.0349 | 0.05978 | 0.09524 | 0.03201 | 0.0553 | 0.01073 | 0.42424 | 0.03076 | 0.14768 | 0.05997 | - |  |  |  |
| **Morocco** | 0.06871 | 0.05517 | 0.08912 | 0.07196 | 0.0803 | 0.11833 | -0.01466 | 0.00061 | 0.03487 | 0.25016 | 0.04421 | 0.17222 | 0.03672 | 0.08004 | - |  |  |
| **Sardinia** | 0.02092 | 0.01836 | -0.00571 | -0.01078 | 0.01073 | -0.00551 | 0.02297 | 0.04166 | -0.02375 | 0.4817 | 0.04449 | 0.10191 | 0.04826 | 0.04433 | 0.07744 | - |  |
| **Portugal** | 0.01114 | 0.01186 | -0.00455 | -0.01419 | 0.00859 | -0.00673 | 0.00745 | 0.0322 | -0.03262 | 0.44496 | 0.02762 | 0.10442 | 0.03494 | 0.03017 | 0.06506 | 0.00286 | - |

**Supplementary Data 3b.** Pairwise F_ST_ values between modern Ibizans and candidate source populations. Modern populations are indicated by black text and Phoenician populations are indicated by red text.

**Supplementary Data 4**


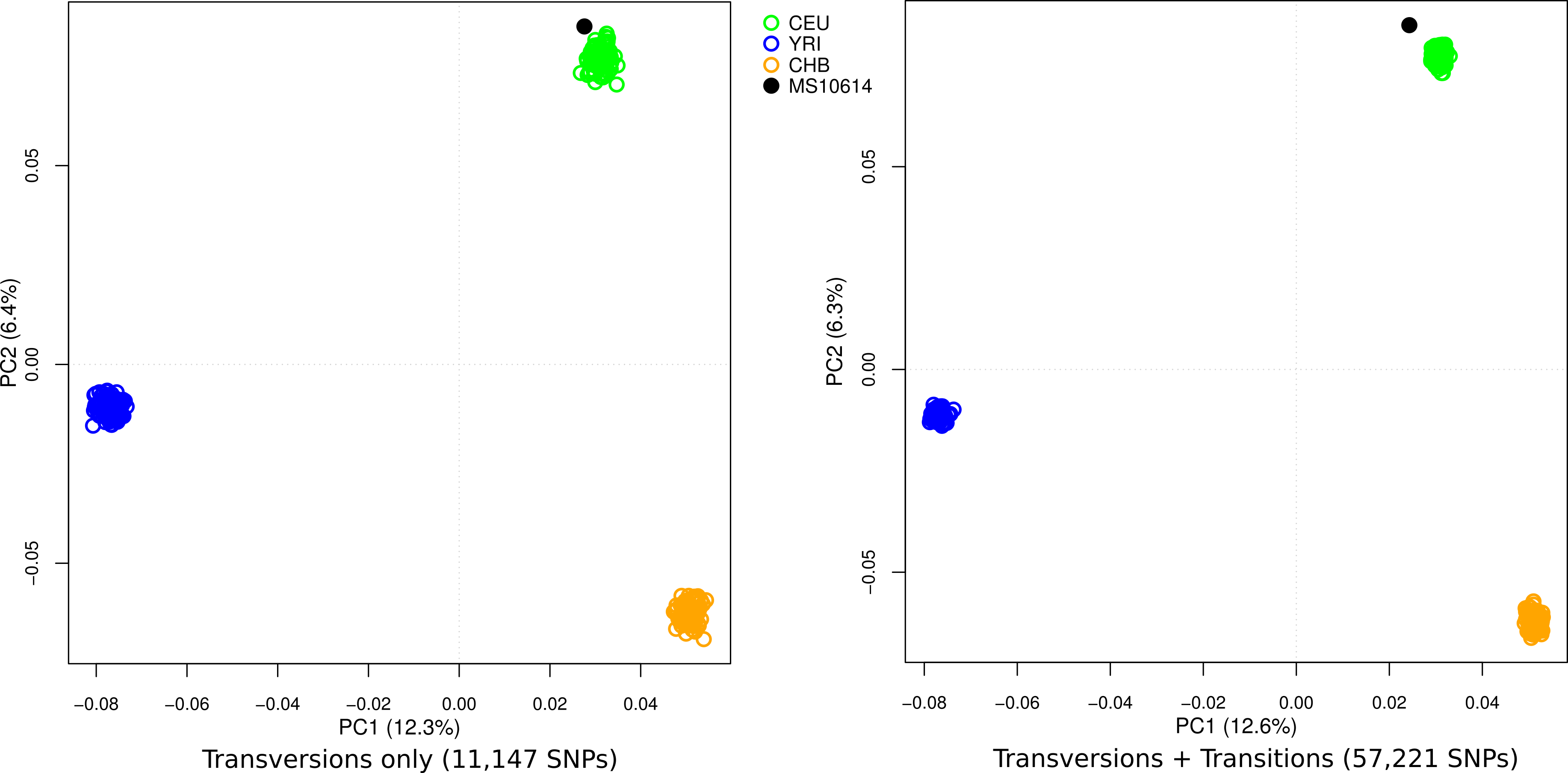


**Supplementary Figure 4a**. Principal Component Analysis (PCA). Projection of sample MS10614 onto the 1000 genomes 3 Worldwide Populations.


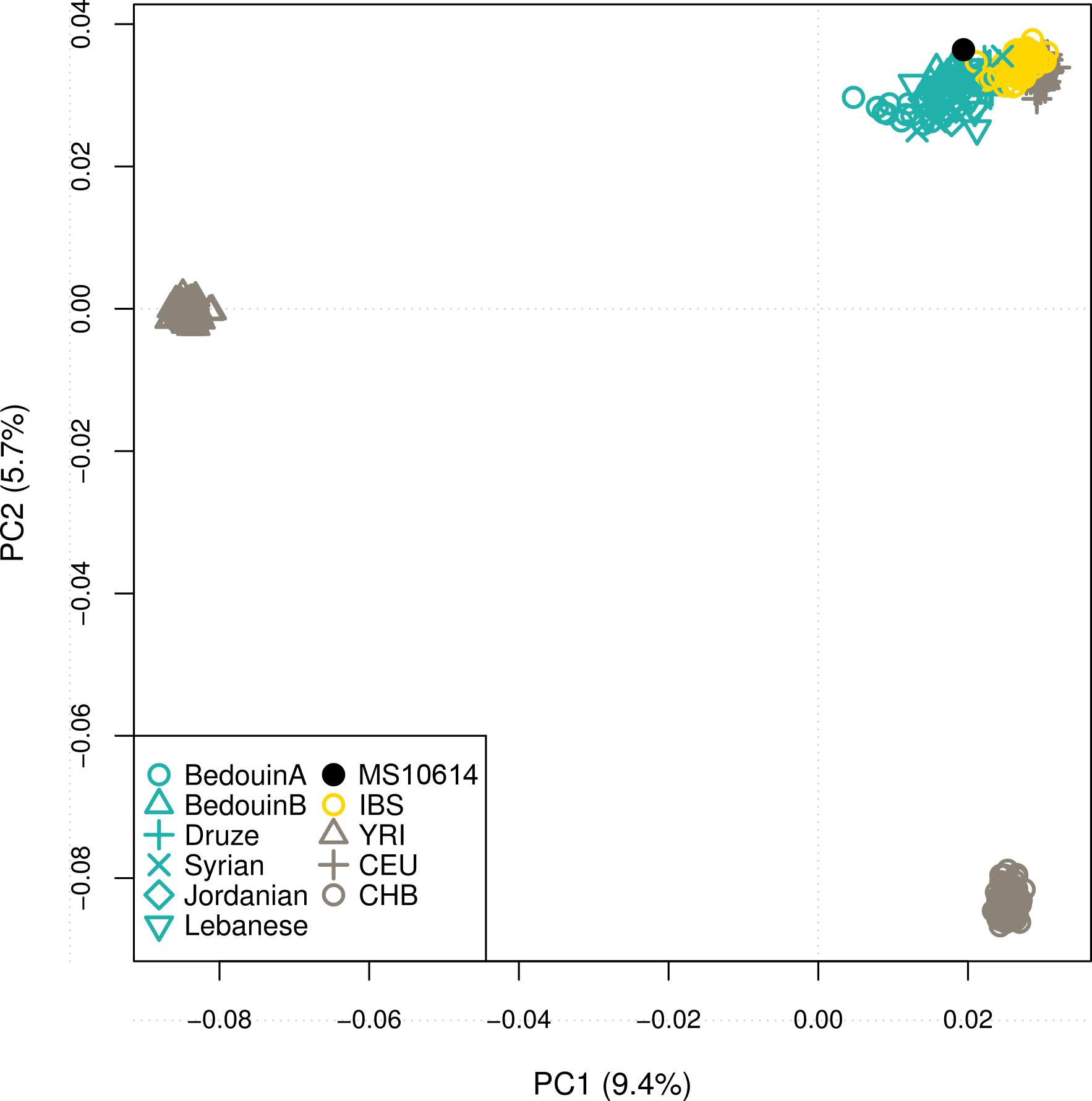


**Supplementary Figure 4b**. Principal Component Analysis (PCA). Projection of MS10614 onto the 1000 genomes 3 Worldwide Populations in addition to other Near Eastern and Ibizan (IBS) populations.


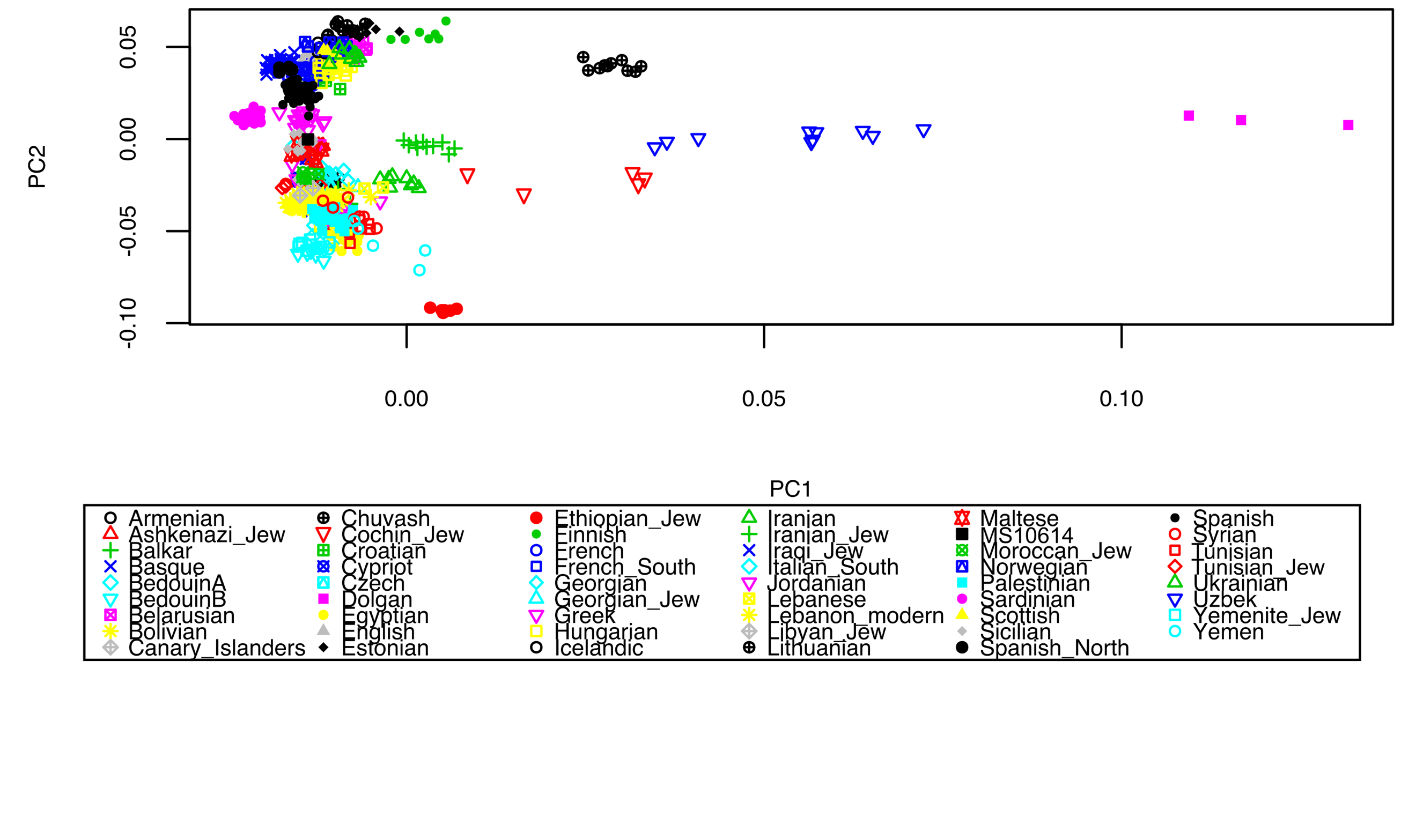


**Supplementary Figure 4c**. Principal Component Analysis (PCA). Projection of MS10614 onto the fully public genotype dataset described in Haak et al. 2015 and available at: https://reich.hms.harvard.edu/datasets


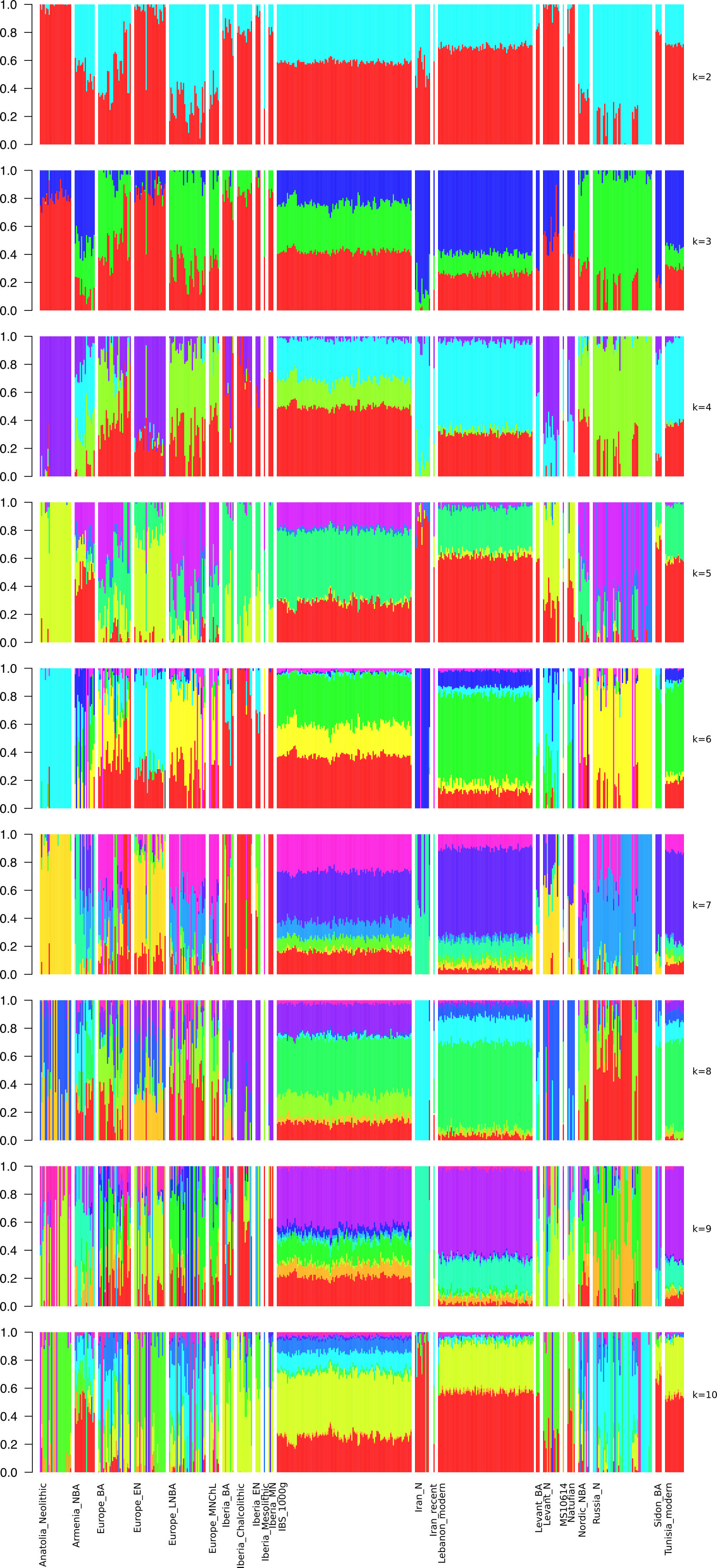


**Supplementary Figure 5.** Complete results from the ADMIXTURE analysis using all samples in the merged data set, displayed for *k*=2 to *k*=10. Ancient samples from the Neolithic/Early Neolithic (N, EN), Chalcolithic, and Bronze Age (BA).

**Supplementary Data 6**


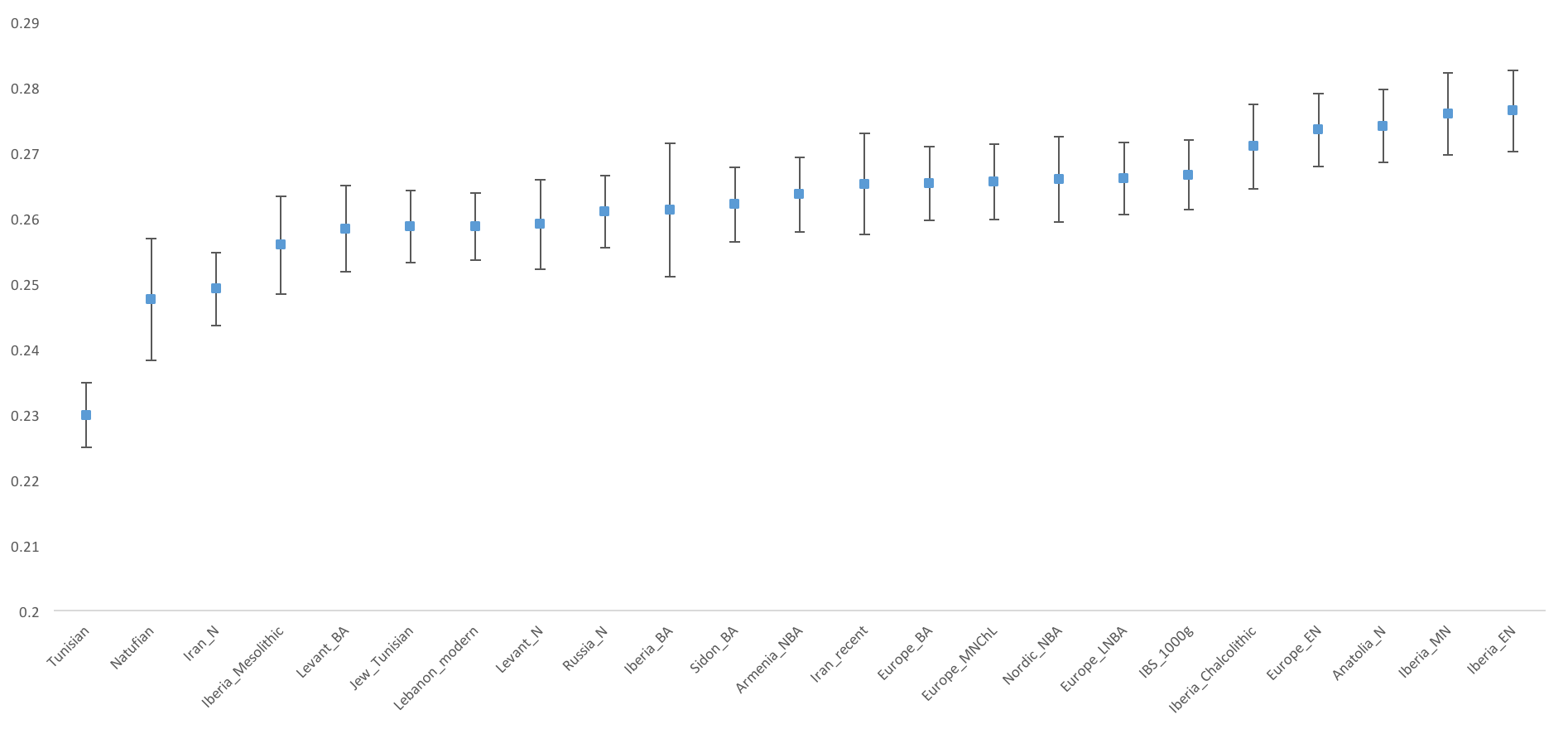


**Supplementary Figure 6a** Outgroup f_3_ statistics measuring shared genetic drift between MS10614 and other ancient and modern populations

**Supplementary Data 6b** D statistics measuring admixture between MS10614 and potential source populations

| **Pop1** | **Pop2** | **Pop3** | **Pop4** | **D-stat** | **Z** | **BABA** | **ABBA** | **# SNPs** |
| --- | --- | --- | --- | --- | --- | --- | --- | --- |
| MS10614 | Iberia_MN | Iberia_Chalcolithic | Mbuti | -0.007661 | -6.485 | 2559 | 2822 | 34426 |
| MS10614 | Iberia_EN | Iberia_Chalcolithic | Mbuti | -0.007393 | -6.249 | 2580 | 2837 | 34752 |
| MS10614 | Europe_EN | Iberia_Chalcolithic | Mbuti | -0.006075 | -5.731 | 2631 | 2844 | 35080 |
| MS10614 | Anatolia_Neolithic | Levant_N | Mbuti | -0.006971 | -5.586 | 2253 | 2467 | 30623 |
| MS10614 | Europe_EN | Levant_N | Mbuti | -0.006507 | -5.195 | 2261 | 2461 | 30623 |
| MS10614 | Europe_MNChL | Iberia_Chalcolithic | Mbuti | -0.005475 | -4.779 | 2688 | 2880 | 35076 |
| MS10614 | Anatolia_Neolithic | Iberia_Chalcolithic | Mbuti | -0.004986 | -4.612 | 2654 | 2829 | 35081 |
| MS10614 | Iberia_EN | Levant_N | Mbuti | -0.006392 | -4.346 | 2225 | 2418 | 30328 |
| MS10614 | Iberia_Chalcolithic | Levant_N | Mbuti | -0.005878 | -4.213 | 2149 | 2319 | 28988 |
| MS10614 | Levant_BA | Levant_N | Mbuti | -0.005877 | -3.729 | 2074 | 2236 | 27531 |
| MS10614 | Iberia_MN | Levant_N | Mbuti | -0.005169 | -3.593 | 2243 | 2398 | 30093 |
| MS10614 | Europe_MNChL | Levant_N | Mbuti | -0.004799 | -3.374 | 2324 | 2471 | 30622 |
| MS10614 | Natufian | Levant_N | Mbuti | -0.00725 | -3.257 | 1209 | 1325 | 15992 |
| MS10614 | Europe_BA | Iberia_Chalcolithic | Mbuti | -0.003241 | -3.024 | 2702 | 2815 | 35077 |
| MS10614 | Iberia_Mesolithic | Iberia_Chalcolithic | Mbuti | -0.003627 | -2.306 | 2311 | 2418 | 29442 |
| MS10614 | Levant_N | Europe_EN | Mbuti | -0.002588 | -2.277 | 2381 | 2461 | 30623 |
| MS10614 | Sidon_BA | Levant_N | Mbuti | -0.002908 | -2.043 | 2364 | 2453 | 30626 |
| MS10614 | Levant_N | Iberia_Chalcolithic | Mbuti | -0.002816 | -2.011 | 2237 | 2319 | 28988 |
| MS10614 | IBS_1000g | Iberia_Chalcolithic | Mbuti | -0.00186 | -1.785 | 2724 | 2789 | 35081 |
| MS10614 | Europe_LNBA | Iberia_Chalcolithic | Mbuti | -0.001938 | -1.747 | 2732 | 2800 | 35081 |
| MS10614 | Iberia_BA | Iberia_Chalcolithic | Mbuti | -0.003309 | -1.575 | 1040 | 1085 | 13573 |
| MS10614 | IBS_1000g | Europe_EN | Mbuti | -0.001158 | -1.448 | 3121 | 3168 | 39951 |
| MS10614 | Iberia_BA | Levant_N | Mbuti | -0.003431 | -1.329 | 908 | 949 | 11982 |
| MS10614 | Armenia_NBA | Levant_N | Mbuti | -0.001725 | -1.277 | 2348 | 2401 | 30501 |
| MS10614 | Europe_BA | Levant_N | Mbuti | -0.001548 | -1.22 | 2345 | 2393 | 30620 |
| MS10614 | Iran_recent | Levant_N | Mbuti | -0.001788 | -0.92 | 1922 | 1967 | 25016 |
| MS10614 | Nordic_NBA | Iberia_Chalcolithic | Mbuti | -0.000988 | -0.776 | 2731 | 2765 | 34644 |
| MS10614 | Iran_recent | Europe_EN | Mbuti | -0.000971 | -0.765 | 2253 | 2281 | 28598 |
| MS10614 | IBS_1000g | Levant_N | Mbuti | -0.000756 | -0.631 | 2359 | 2382 | 30626 |
| MS10614 | Nordic_NBA | Europe_EN | Mbuti | -0.000596 | -0.595 | 3071 | 3095 | 38968 |
| MS10614 | Iran_recent | Iberia_Chalcolithic | Mbuti | -0.000858 | -0.551 | 2181 | 2205 | 27649 |
| MS10614 | Europe_LNBA | Levant_N | Mbuti | -0.00052 | -0.409 | 2377 | 2393 | 30625 |
| MS10614 | Lebanon_modern | Levant_N | Mbuti | -0.000394 | -0.322 | 2383 | 2395 | 30626 |
| MS10614 | Levant_BA | Europe_EN | Mbuti | -0.000306 | -0.29 | 2535 | 2545 | 32193 |
| MS10614 | Sidon_BA | Europe_EN | Mbuti | -0.000126 | -0.136 | 3178 | 3184 | 39951 |
| MS10614 | Nordic_NBA | Levant_N | Mbuti | -0.000038 | -0.025 | 2349 | 2351 | 30327 |
| MS10614 | Levant_BA | Iberia_Chalcolithic | Mbuti | 0.000035 | 0.027 | 2420 | 2419 | 30643 |
| MS10614 | Jew_Tunisian | Levant_N | Mbuti | 0.00013 | 0.106 | 2385 | 2381 | 30626 |
| MS10614 | Armenia_NBA | Iberia_Chalcolithic | Mbuti | 0.000129 | 0.113 | 2753 | 2748 | 34913 |
| MS10614 | Natufian | Iberia_Chalcolithic | Mbuti | 0.000243 | 0.127 | 1360 | 1356 | 17130 |
| MS10614 | Natufian | Europe_EN | Mbuti | 0.000272 | 0.169 | 1431 | 1426 | 17989 |
| MS10614 | Iberia_Mesolithic | Levant_N | Mbuti | 0.000888 | 0.463 | 1997 | 1974 | 25250 |
| MS10614 | Russia_N | Iberia_Chalcolithic | Mbuti | 0.000607 | 0.561 | 2786 | 2765 | 35081 |
| MS10614 | Iran_N | Levant_N | Mbuti | 0.001116 | 0.776 | 2396 | 2362 | 30223 |
| MS10614 | Lebanon_modern | Europe_EN | Mbuti | 0.001129 | 1.403 | 3184 | 3138 | 39951 |
| MS10614 | Russia_N | Levant_N | Mbuti | 0.001816 | 1.464 | 2412 | 2356 | 30626 |
| MS10614 | Sidon_BA | Iberia_Chalcolithic | Mbuti | 0.001802 | 1.497 | 2808 | 2744 | 35081 |
| MS10614 | Russia_N | Europe_EN | Mbuti | 0.001251 | 1.507 | 3193 | 3143 | 39951 |
| MS10614 | Jew_Tunisian | Iberia_Chalcolithic | Mbuti | 0.001657 | 1.551 | 2789 | 2731 | 35081 |
| MS10614 | Jew_Tunisian | Europe_EN | Mbuti | 0.001333 | 1.632 | 3180 | 3126 | 39951 |
| MS10614 | Lebanon_modern | Iberia_Chalcolithic | Mbuti | 0.002081 | 1.967 | 2803 | 2730 | 35081 |
| MS10614 | Iran_N | Iberia_Chalcolithic | Mbuti | 0.003608 | 3.075 | 2809 | 2685 | 34508 |
| MS10614 | Iran_N | Europe_EN | Mbuti | 0.003621 | 3.893 | 3140 | 3001 | 38294 |
| MS10614 | Tunisian | Levant_N | Mbuti | 0.008474 | 6.872 | 2561 | 2301 | 30626 |
| MS10614 | Tunisian | Iberia_Chalcolithic | Mbuti | 0.010544 | 9.716 | 3000 | 2630 | 35081 |
| MS10614 | Tunisian | Europe_EN | Mbuti | 0.010332 | 12.484 | 3425 | 3012 | 39951 |
